# Metrics and Evaluation Tools for Patient Engagement in Healthcare Organization- and System-Level Decision-Making: A Systematic Review

**DOI:** 10.15171/ijhpm.2018.43

**Published:** 2018-05-16

**Authors:** Vadim Dukhanin, Rachel Topazian, Matthew DeCamp

**Affiliations:** ^1^Department of Health Policy and Management, Johns Hopkins Bloomberg School of Public Health, Baltimore, MD, USA.; ^2^National Journal, Washington, DC, USA.; ^3^Johns Hopkins Berman Institute of Bioethics, Baltimore, MD, USA.; ^4^Division of General Internal Medicine, Department of Medicine, Johns Hopkins School of Medicine, Baltimore, MD, USA.

**Keywords:** Patient Engagement, Patient Participation, Health Systems, Health Planning, Organizational Decision-Making

## Abstract

**Background:** Patient, public, consumer, and community (P2C2) engagement in organization-, community-, and systemlevel healthcare decision-making is increasing globally, but its formal evaluation remains challenging. To define a taxonomy of possible P2C2 engagement metrics and compare existing evaluation tools against this taxonomy, we conducted a systematic review.

**Methods:** A broad search strategy was developed for English language publications available from January 1962 through April 2015 in PubMed, Embase, Sociological Abstracts, PsycINFO, EconLit, and the gray literature. A publication was excluded if: (1) the setting was not healthcare delivery (ie, we excluded non-health sectors, such as urban planning; research settings; and public health settings not involving clinical care delivery); (2) the P2C2 engagement was episodic; or (3) the concept of evaluation or possible evaluation metrics were absent. To be included as an evaluation tool, publications had to contain an evaluative instrument that could be employed with minimal modification by a healthcare organization.

**Results:** A total of 199 out of 3953 publications met exclusion and inclusion criteria. These were qualitatively analyzed using inductive content analysis to create a comprehensive taxonomy of 116 possible metrics for evaluating P2C2 engagement. 44 outcome metrics were grouped into three domains (internal, external, and aggregate outcomes) that included six subdomains: impact on engagement participants, impact on services provided by the healthcare organization, impact on the organization itself, influence on the broader public, influence on population health, and engagement cost-effectiveness. The 72 process metrics formed four domains (direct process metrics; surrogate process metrics; aggregate process metrics; and preconditions for engagement) that comprised sixteen subdomains. We identified 23 potential tools for evaluating P2C2 engagement. The identified tools were published between 1973-2015 and varied in their coverage of the taxonomy, methodology used (qualitative, quantitative, or mixed), and intended evaluators (organizational leaders, P2C2 participants, external evaluators, or some combination). Parts of the metric taxonomy were absent from all tools.

**Conclusions:** By comprehensively mapping potential outcome and process metrics as well as existing P2C2 engagement tools, this review supports high-quality P2C2 engagement globally by informing the selection of existing evaluation tools and identifying gaps where new tools are needed.

**Systematic Review Registration:** PROSPERO registration number CRD42015020317.

## Introduction

Ensuring that individuals and communities are engaged in healthcare decision-making is now widely regarded as a requirement of patient centered care. One part of engagement, enshrined as a right of all people in the 1978 Declaration of Alma-Ata,^1^ requires engaging patients not only in their own individual medical decisions but also in the design and implementation of healthcare services.^2,3^ This type of engagement is occurring globally. In the United States, some jurisdictions require patient and family advisory councils to inform individual hospital governance,^4^ major healthcare reform efforts require patient representation on governance boards,^5^ and many healthcare organizations and systems see patient engagement as the right thing to do (a recurring theme since at least the 1970s).^6-9^ In the United Kingdom,^10-12^ Canada,^13-15^ Australia,^16,17^ and New Zealand,^18^ there has also been systematic public involvement in healthcare decision-making via regional or local health advisory councils, committees, boards or citizen juries. Public engagement in health sector priority-setting has also been mandated or promoted in low- and middle-income countries.^19-22^

Despite increasing attention to patient engagement in the design and implementation of healthcare services, there is both a greater need for formal engagement evaluation^23^ and little agreement on how to do so. Existing how-to guides suggest that patient engagement can contribute positively to health outcomes, reduce unnecessary costs, or increase trust in healthcare organizations,^24,25^ but real evidence of these impacts, according to literature reviews, is lacking.^26-28^ A 2010 review of engagement in healthcare policies and programs concluded that existing evaluations narrowly focus on short-term impact and proxy or surrogate measures of perceived impact.^29^

Given the evidence gap and the need to update existing reviews, we conducted a systematic review of metrics to evaluate patient, public, consumer and community (P2C2) engagement in organization-, community-, and system-level healthcare decision-making. We defined P2C2 engagement as a continuous systematic effort to incorporate the needs, values, and preferences of the P2C2 engagement participants into decision-making. Though there may be differences between patients, the public, consumers, and the community in concept, in practice healthcare organizations may use the terms interchangeably when engaging those stakeholders in decision-making, and our focus was upon what is common to all those engagement activities – namely, the goal of incorporating those stakeholders’ needs, values and preferences. In those activities, P2C2 engagement participants are involved as stakeholder representatives of their constituents, rather than as individuals. This review focuses on engagement in organization-, community-, and system-level healthcare decision-making as distinct from patient engagement in their individual personal medical decisions.^2,3^ Our objectives were (1) to create a taxonomy of possible P2C2 engagement evaluation metrics using an inductive qualitative analysis of the literature and then (2) to compare existing P2C2 engagement evaluation tools against this taxonomy.

## Methods

### Identification of Publications

The nature of our subject matter required a broad search strategy within the published and gray literature. We conducted a search for publications available in English from January 1, 1962 through April 20, 2015 in PubMed, Embase, Sociological Abstracts, PsycINFO, and EconLit with Full Text. Terminology surrounding P2C2 “engagement” may be imprecise; therefore, we designed our search to cast a wide net, then relied upon our inclusion/exclusion criteria to identify target publications. Search terms were identified via consultations with an informationist and through an iterative process of search yield analyses. The final search included three concept blocks: (1) P2C2 engagement; (2) decision-making; and (3) healthcare planning.

The first block included 125 search terms, which combined (*i*) “patient,” “consumer,” “stakeholder,” “community,” “public,” and related words with (*ii*) “engagement,” “participation,” “involvement,” “representation,” “advocacy,” and related words. The second block included 40 search terms, such as “decision-making,” “clinical governance,” “policy-making,” “governing board,” “participatory management,” and related words. The third block included 61 search terms, which combined (*i*) “healthcare,” “health,” and “medical” with (*ii*) “system,” “organization,” “planning,” “priority,” and related words. For all three concept blocks we included corresponding controlled vocabulary items relevant to the particular database. To be included, a publication had to contain at least one term or controlled vocabulary item from each block. Full details of the search strategy are included in online Supplementary file 1. After combining all database search results and removing duplicates, this search yielded 3953 possible publications.

Gray literature publications were identified throughout May 2015–July 2016 from sources identified during review of the published literature and via a similar search strategy of select websites. The 32 targeted websites of relevant national and international organizations are included in online Supplementary file 2. The gray literature search yielded 97 additional possible publications.

Lastly, during our full text review, in reference lists we identified 44 additional possible publications which were reviewed in full-text. This systematic review was included on May 7, 2015 in the International Prospective Register of Systematic Reviews (PROSPERO), registration number CRD42015020317.

### Criteria for Selection

**Figure 1:**
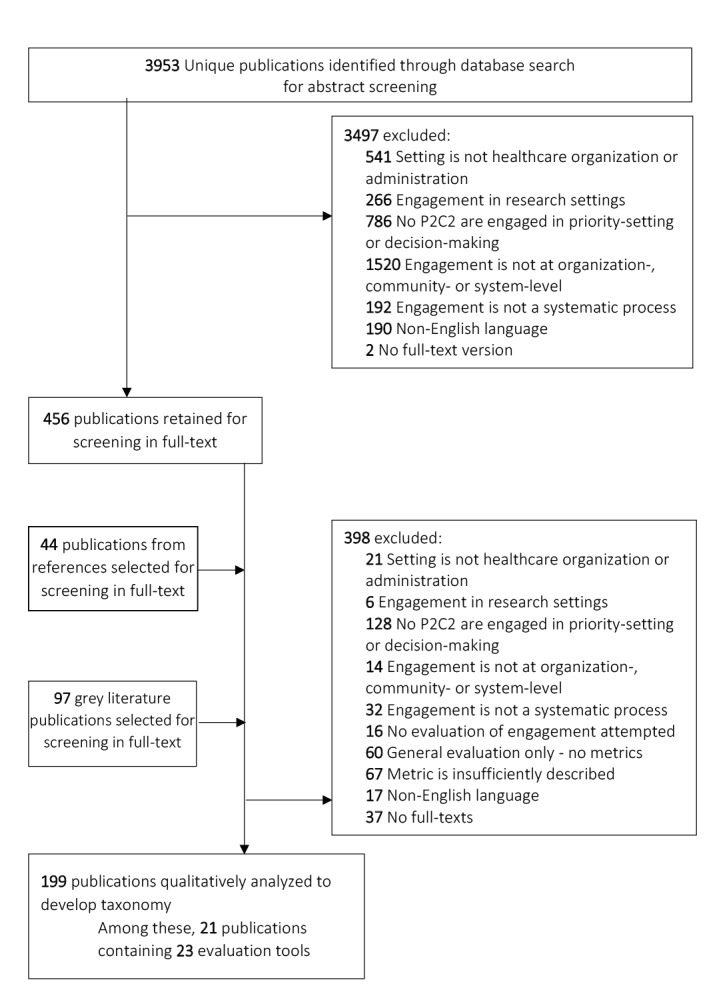
Flow Diagram Describing Publication Selection.

Figure 1 describes the publication selection process. We excluded publications whose language was not in English or whose publication type did not provide a full-text version. We included publications if their setting was healthcare administration or healthcare organization in clinical settings. A publication was excluded if the setting was (1) outside the healthcare sector (eg, urban planning, environmental services, and education sectors, among others, would be excluded); (2) research; or (3) outside of clinical care delivery (eg, public health and/or health promotion programs). Moreover, to be included, publications needed to contain a description of P2C2 engagement in decision-making at the organizational-, community-, or system-level; thus, we excluded publications describing engagement exclusively related to personal medical decisions. Since we also required engagement to be a continuous systematic process (as opposed to episodic or one-off engagement), we excluded publications that did not reference more than one instance of engagement.

First, two reviewers (either VD and RT or VD and MD) independently screened abstracts by applying these exclusion and inclusion criteria. Inter-reviewer disagreement was resolved through discussion involving the third reviewer (either MD or RT). If inclusion or exclusion could not be determined based on an abstract alone, the publication was retained for full-text review. Next, the same selection procedure and criteria were applied for full-text review. Final inclusion eligibility was dependent on the presence of evaluation of engagement, either via a sufficiently detailed definition of a possible measure or via a discrete measure itself. In total 199 publications were retained for this review.

Among these 199, reviewers noted any publications that included an evaluation tool. We defined a tool as a questionnaire, survey, or other evaluative device that a healthcare organization or system could be used with no or minimal modification to evaluate P2C2 engagement efforts. We identified 21 publications containing 23 evaluation tools.

### Developing a Taxonomy of Metrics From the Literature

When reviewing the 199 publications, reviewers independently annotated each publication for the presence of evaluation metrics. This was done descriptively, via an editing style of qualitative content analysis.^30-32^ A reviewer would read the publication in its entirety, highlighting text descriptions of possible evaluation metrics. The reviewer would then inductively apply a descriptive annotation (or “code”) of what the evaluation metric intended to measure. To illustrate, if a publication referenced measuring P2C2 participants’ “improved knowledge of the healthcare system,” this would be descriptively annotated as “knowledge” metric. Next, thematic analysis was used to combine and group similarly themed annotations into one list. Finally, using the accepted distinction between process and outcome metrics in healthcare evaluation, the research team (VD, RT, MD) discussed and reorganized the list into a taxonomy of possible metrics, grouping thematically-related metrics into domains and subdomains of process and outcome categories.

### Applying the Taxonomy to Evaluation Tools

Subsequently, VD and MD, in collaboration, conducted a second qualitative analysis of the included evaluation tools using NVivo Plus (QSR International Pty Ltd, Version 11, 2016). Each tool’s questions or survey items were coded according to the taxonomy: firstly, (1) into the process or outcome category; secondly (2) into the metric subdomain, and thirdly (3) as a specific metric within that subdomain. If a question or item was non-specific and could not be coded in steps (2) or (3), it was coded only into the broader category or subdomain. Items could be coded more than once, and coding frequencies were tracked. Based on the resulting frequency histogram, frequencies were transformed into a categorical scale (categories: 1-3 instances, 4-14 instances, or 15 or more instances of coding). Evaluation tools were also descriptively characterized by country, publication date, method of evaluation (quantitative, qualitative or mixed), intended evaluators (leaders, P2C2 representatives, both leaders and representatives, or external evaluators) and setting (healthcare program, individual hospital, healthcare system agency and others). Due to the nature of extracted data, standard procedures to assess quality of studies reporting the metrics or tools were not applicable.

## Results

### Overview of the Included Publications

We identified 199 eligible publications, which were qualitatively analyzed to create a taxonomy of possible metrics of P2C2 engagement in healthcare organization-, community-, and system-level decision-making. Of these, 21 presented 23 distinct evaluation tools.^33-53^ The characteristics of these evaluation tools are in Table 1 (the remaining 178 publications are listed in the online Supplementary file 3).

**Table 1:** Listing of Identified Evaluation Tools and their Basic Characteristics

**Tool Name (If Applicable), Authors**	**Country**	**Year**	**Setting**	**Method of Evaluation**	**Brief Description**	**Who Fills Out Evaluation**
Metsch and Veney^33^	USA	1973	Individual hospital	Quantitative	Scoring tool for meeting minutes that assigns weighted categories of interaction for each consumer recommendation.	External evaluators
Steckler and Dawson^34^	USA	1978	Health Systems Agency	Quantitative	38 questions in five items/indices and interview data. Adapted from^i-iv^.	Leaders and P2C2 representatives
Rifkin, Muller, and Bichmann^35^	Nepal	1988	Healthcare program	Quantitative	Qualitative data scored 1 to 5 in five dimensions using a ranking table.	External evaluators
Schmidt and Rifkin^36^	Tanzania	1996	Healthcare program	Quantitative	Same tools as #3 applied in a different country.	External evaluators
Consumer Participation Questionnaire, Kent and Read^37^	New Zealand	1998	Mental health services	Mixed method	Yes/No, Likert scale, and discrete choice items.	Leaders
El Ansari and Phillips^38^	South Africa	2001	Healthcare program	Quantitative	7-point Likert scale covering eight dimensions. Derived from sources evaluating engagement outside healthcare.	Leaders and P2C2 representatives
Partnership self-assessment survey, Shortell et al^39^	USA	2002	Diverse	Quantitative	5-point Likert scale items. Four components are measured using 1-5 items.	Leaders and P2C2 representatives
Halliday et al^40^	UK	2004	Diverse	Mixed method	4-point Likert scale (covering nine dimensions) and open-ended questions. Derived from^v-vi^.	Leaders and P2C2 representatives
Jarrett and Patient Involvement Unit^41^	UK	2004	Guideline development group	Mixed method	5-point Likert scale and interview questions in open-ended fashion, both evaluating the same aspects.	Leaders and P2C2 representatives
A Hospital Self-Assessment Inventory. Institute for Family-Centered Care^42^	USA	2004	Individual hospital	Mixed method	5-point Likert scale, and 3-point rating system and open-ended notes.	Leaders and P2C2 representatives
Well Connected, South et al^43^	UK	2005	Healthcare program	Quantitative	10-point scale covering six dimensions based on three general scoring criteria. Drawn from tool #3 and^vi-viii^.	Leaders and P2C2 representatives
Grant^44^	Canada	2007	Mental health services	Mixed method	Yes/No questions, Likert scale questions, and multiple choice items. Modified by adding questions to tool #4.	Leaders
Evaluation Form, Health and Social Care Regulatory Forum^45^	Ireland	2009	Diverse	Mixed method	5-point Likert and open-ended questions. Derived from^ix^.	Leaders
Draper et al^46^	Djibouti, Honduras, and Nepal	2010	Healthcare program	Quantitative	5-point scale scoring five factors (tool provides example descriptions for 1-, 3-, and 5-point scores). Modified #3, by replacing two domains.	External evaluators
PFAC Annual Report Template, Consumer Health Quality Council HCFA, Massachusetts^47^	USA	2012	Individual hospital	Qualitative	Open-ended items and multiple-choice questions.	Leaders
Consumer Health Quality Council Review Instrument for 2011 Reports, Consumer Health Quality Council HCFA, Massachusetts^47^	USA	2012	Individual hospital	Mixed method	Open-ended, multiple choice and Yes/No questions.	External evaluators
National Institute for Children's Health Quality^48^	USA	2012	Diverse	Mixed method	3-point Likert scale questions with “free-text” field and a set of 5-point Likert scale questions. Derived from *Essential Allies.*^25^	Leaders
HCFA Recommended 2013 PFAC Annual Report Template HCFA, Massachusetts^49^	USA	2014	Individual hospital	Mixed method	Multiple-choice and open-ended questions	Leaders and P2C2 representatives
HCFA 2013 PFAC Report Review Tool, HCFA, Massachusetts^49^	USA	2014	Individual hospital	Mixed method	Open-ended, multiple choice and Yes/No questions.	External evaluators
PFAC Council Evaluation. Brigham and Women’s Hospital Center for Patients and Families^50^	USA	2014	Individual hospital	Mixed method	5-point Likert scale and open-ended questions.	Leaders and P2C2 representatives
PPEET, Participant questionnaire, v. 1.0. Abelson and PPEET Research-Practice Collaborative^51^	Canada	2015	Diverse	Mixed method	5-point Likert scale plus open-ended questions.	P2C2 representatives
PPEET, Organization questionnaire, v. 1.0. Abelson and PPEET Research-Practice Collaborative^51^	Canada	2015	Diverse	Mixed method	5-point Likert scale plus open-ended questions.	Leaders
CCP evaluation framework, CCP Steering Committee^52^	USA	2015	Medicaid Managed Care	Quantitative	Review of program websites and documents with Yes/No checkboxes.	External evaluators
National ACO Patient Activation and Engagement Survey, Shortell et al^53^	USA	2015	ACO	Quantitative	1 to 9 Likert scale and Yes/No items. Part of the National Survey of ACOs.	Leaders

Abbreviations: ACO, accountable care organization; CCP, Consumer Confidence Project; HCFA, Health Care For All; P2C2, patient, public, consumer and community; PFAC, Patient and Family Advisory Council; PPEET, Public and Patient Engagement Evaluation Tool. References that appear only in Table 1: i. Beck A, Bishop P. *The Consumer Support Group: A Report to the Board of Trustees, Capitol Area Comprehensive Health Planning Association*. Lansing, Michigan, 1973. ii. Douglass C. Representation patterns in community health decision-making. *J Health Soc Behav* 1973;14(1):80-86. doi:10.2307/2136939. iii. Douglass C. Effect of provider attitudes in community health decision-making. Med Care 1973;11(2):135-144. iv. Douglass C. Health Services Planning in the Urban Ghetto: A Comparative Analysis of Eight Model Cities Programs [dissertation]. University of Michigan School of Public Health, Ann Arbor, 1971. v. Hardy B, Hudson B, Waddington E. *What Makes a Good Partnership? A Partnership Assessment Tool*. Leeds: Nuffield Institute for Health, Community Care Division, 2000. vi. World Health Organization/Health Education Board for Scotland. *Verona Benchmark: Guide to the Assessment of Good Practice within Partnership Working.* 2000. vii. Yorkshire Forward. *Active Partners. Benchmarking Community Participation in Regeneration*. Leeds: Yorkshire Forward, 2000. viii. Funnell R, Olfield K, Speller V. *Towards Healthier Alliances: A Tool for Planning, Evaluating and Developing Healthy Alliances*. London: Health Education Authority, Wessex Institute for Health, 1995. ix. Irish Society for Quality and Safety in Healthcare and Health Care Informed. *Now We’re Talking: A practical toolkit for public and patient involvement in healthcare*. Dublin: Irish Society for Quality and Safety in Healthcare, 2009.

Identified tools were published between 1973 and 2015. Twelve out 23 were implemented in the United States, three in Canada, three in the United Kingdom, two in Nepal, and the rest were implemented once in other low-middle-income (Djibouti, Honduras, South Africa, or Tanzania) or high-income countries (Ireland or New Zealand). Thirteen tools used mixed method evaluation, six only quantitative evaluation and one only qualitative evaluation. The evaluation was filled out by patient, public, consumer, and community (P2C2) representatives in one tool, by organization leaders in seven tools, by both representatives and leaders in nine tools, and by external evaluators in six tools. The tools were used in diverse settings, from individual hospitals to health systems and programs.

### Taxonomy of Metrics

**Figure 2:**
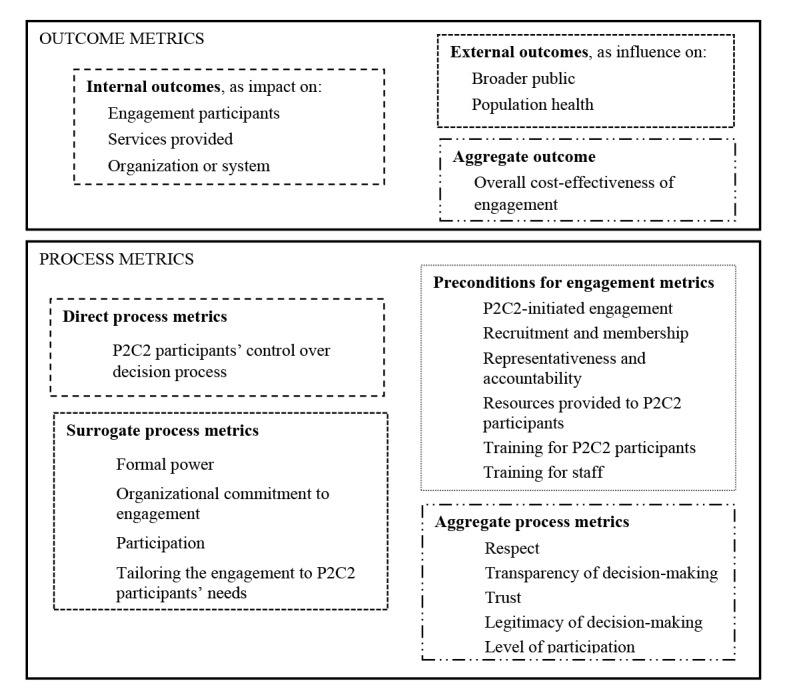
Taxonomy of Metrics: Metrics Categories, Domains and Subdomains. For the full taxonomy with listed metrics forming each subdomain, please refer to online Supplementary file 4.

We organized metrics of P2C2 engagement into two main categories: outcome metrics and process metrics.^54^ Within each category we identified subdomains which we subsequently clustered into domains based on thematic relatedness. Figure 2 illustrates the taxonomy’s domains and subdomains within the categories. Please refer to online Supplementary file 4 for the full taxonomy with metrics forming each subdomain listed.

#### Outcome Metrics

We identified 44 unique outcome metrics forming six distinct subdomains. Further, we clustered related subdomains into three domains: (*i*) internal outcomes, (*ii*) external outcomes, and (*iii*) aggregate outcomes. ‘Internal outcomes’ were those subdomains containing metrics most relevant to, and evaluated within, a healthcare organization or system. This domain included three subdomains: impact on engagement participants themselves (eg, improved knowledge among P2C2 participants), impact on the services provided by the organization or system (eg, improved quality or decreased utilization of services), and impact on the organization or system (on its policies, procedures or resources, eg, redesign of staff roles, or staff training policies, or expanding its patient engagement program).

By contrast, ‘external outcomes’ were those subdomains containing metrics most relevant beyond the organization or system itself and requiring evaluation outside the organization. This group included two subdomains: influence on the broader public (eg, strengthened public support of the organization) or on population health generally (eg, decreased health inequalities).

Finally, we kept cost effectiveness of engagement as a separate ‘aggregate outcome’ domain since the overall cost-effectiveness from the standpoint of the healthcare organization or system could take into account all changes (positive or negative) throughout all subdomains.

#### Process Metrics

We identified 72 unique process metrics forming 16 subdomains. We clustered related subdomains into four domains: (*i*) direct process metrics; (*ii*) surrogate process metrics; (*iii*) preconditions for engagement metrics; and (*iv*) aggregate process metrics.

‘Direct process metrics’ included subdomains that describe the degree of real control that P2C2 participants have over the decision-making process. For instance, whether P2C2 participants set the agenda, have well-defined roles, or are able to evaluate and revise the decision process itself are all metrics that could be considered as those of direct control over the process. Other examples of such metrics are whether P2C2 participants are involved in the decision process since its first stages, or P2C2 participants are included in all types of decisions and activities, or P2C2 participants are allowed the opportunity to finalize the decisions.

By contrast, the domain of ‘surrogate process metrics’ does not evaluate direct control over decision-making. Instead, surrogate process metrics describe formal attributes of the process. For instance, whether P2C2 participants hold certain formal positions within the organization, have veto power, or are financially independent of the organization are all metrics of formal attributes that could correlate with direct process control. Attendance of the meetings by P2C2 participants is another example of a surrogate metric in the participation subdomain. Surrogate process metrics also included metrics related to the organizational commitment to engagement, such as the presence of a formal declaration of support for engagement, and metrics identifying whether engagement is tailored to P2C2 participants’ needs or beliefs.

‘Preconditions for engagement metrics’ included subdomains of factors necessary for engagement.^55^ Relevant subdomains included metrics covering the type of recruitment process used to identify P2C2 participants, whether resources (eg, parking, transportation, and/or meals) are provided to the P2C2 participants, whether training is provided to P2C2 participants or organizational staff, P2C2 participants’ representativeness and accountability to their constituents, and whether engagement was initiated by P2C2. For instance, under the subdomain of representativeness, metrics could evaluate whether P2C2 participants are representative of their relevant constituency (eg, a disease group), democratically represent a broader community, or explicitly represent minority, vulnerable or marginalized groups.

Finally, ‘aggregate process metrics’ included subdomains of metrics that evaluate cross-domain aspects of engagement and provide an overall summary assessment as a result. For instance, metrics of trust draw upon different aspects of the preconditions, surrogate measures, and direct measures of the engagement process.

### Results of Coding Evaluation Tools According to the Taxonomy

#### Outcome Metrics

Among 23 identified tools (introduced in Table 1), 13 included at least one question or item coded as an outcome of P2C2 engagement (Table 2). The entire taxonomy contains 44 unique outcome metrics; 22 of these were identified in at least one question or item within a tool. Internal outcomes were more frequently coded than external outcomes.

**Table 2:** Coding Frequencies of Outcome Metrics in the Taxonomy per Evaluation Tool

**Metric**		** Tool Number (see Table 1) **												
		2^34^	3^35,36^	5^38^	6^39^	7^40^	8^41^	9^42^	12^45^	14^47^	17^49^	19^50^	20^51^	21^51^
* Outcome metric		√	√	√√	√√√	√	√√	√	√	√	√	√√	√√	√√
**Internal outcomes metrics**	^**^ ** Impact on engagement participants’**			√√	√√	√	√					√√	√√	
	Knowledge			√	√							√	√	
	Skills			√	√									
	Empowerment			√								√		
	Satisfaction			√	√							√	√	
	Trust					√							√	
	^**^ ** Impact on services provided**			√	√					√	√			√
	Efficiency and cost-effectiveness of services				√									
	Service availability			√	√									
	Services quality and safety				√					√	√			
	Services responsiveness to needs				√									
	Utilization of services				√									
	^**^ ** Impact on organization or system**			√√	√√√		√		√					√√
	Accountability of organization to P2C2 served				√									
	Staff views on engagement						√							√
	Formal (written) organization or system policies						√							
	Explicit change to organization or system process of decision-making													√
	Additional connections or partnerships with other groups or organizations			√	√									√
	Funding and resources availability			√	√									
	Visibility of organization				√									
**External outcomes metrics**	^**^ **Influence on broader public’s**			√										√
	Awareness or knowledge of health issues			√										
	Support of the organization or system			√										√
	^**^ **Influence on population health**		√	√	√									
	Level of health inequalities		√											
	Population health status				√									
**Aggregate outcome: Overall cost-effectiveness of engagement**					√									
Filled out by: External Evaluator			E											
^***^Leader		L		L	L	L	L	L	L	L	L	L		L
^***^P2C2 representative		R		R	R	R	R	R			R	R	R	

Abbreviation: P2C2; patient, public, consumer and community. KEY: √ 1-3 instances, √√ 4-14 instances, √√√ 15 or more instances of coding. ^*^ Row includes instances of items coded only into the outcome metric category when further specification was not possible. ^**^ Row include instances of items coded only into the subdomain when further specification was not possible. ^***^ Individual coded items could be asked of only leaders, only representatives or both; data shown are for tool as whole. Metrics absent from all evaluation tools include, by metric subdomain: - Impact on engagement participants’: (1) views; (2) confidence and self-esteem; (3) sense of ownership. - Impact on services provided: (1) number of complaints on services; (2) sustainability of the services; (3) user experiences with services. - Impact on organization or system: (1) presence of racism in system; (2) informal (unwritten) organization or system procedures; (3) staff recruitment; (4) staff training; (5) level of public reporting; (6) number of local employment positions supported by organization; (7) organization ability to adapt to operative environment; (8) scale of engagement program by organization; (9) redesign of staff roles; (10) staff satisfaction; (11) sustainability of engagement initiative; (12) diversity of funding sources. - Influence on broader public’s: (1) capacity for future involvement in the organization by the community; (2) level of control over decisions made by the organization or system; (3) involvement as part of social change outside the organization; (4) stigmatization of others.

Individual tools varied in their coverage of outcome metrics, ranging from one item addressing a general outcome measure to 32 coding instances when a tool’s questions or items included all six outcome metric subdomains. Six tools included four or more instances of coded outcome metrics. Among the five of these containing 4-14 coding instances, internal outcomes were predominant. All six were designed to be filled out by both leaders and P2C2 representatives (including two separately coded tools that comprise one inventory^51^ ).

#### Process Metrics

All 23 identified tools included at least one question or item coded as a process metric of engagement (Table 3). Fifty-six of the 72 unique process metrics described in the taxonomy were present in at least one tool. Direct process metrics evaluating P2C2 control over decision process were identified in every tool. Surrogate process metrics addressing organizational commitment to engagement and participation as well as metrics of preconditions for engagement (ie, resources provided to P2C2 participants), were the most frequent process metrics.

**Table 3:** Coding Frequencies of Process Metrics in the Taxonomy Per Evaluation Tool

**Metric**		**Tool Number (see Table 1)**																						
		1^33^	2^34^	3^35,36^	4^37^	5^38^	6^39^	7^40^	8^41^	9^42^	10^43^	11^44^	12^45^	13^46^	14^47^	15^47^	16^48^	17^49^	18^49^	19^50^	20^51^	21^51^	22^52^	23^53^
^*^ **Process metric**		**√√**	**√√**	**√√**	**√√**	**√√**	**√√√**	**√√**	**√√√**	**√√√**	**√**	**√√**	**√√√**	**√**	**√√√**	**√√**	**√√**	**√√√**	**√√**	**√√√**	**√√**	**√√**	**√√**	**√**
**Direct process metrics**	^**^ **P2C2 participants’ control over decision process:**	√	√√	√	√	√	√	√√	√√√	√√√	√	√	√√	√	√	√	√	√√	√	√	√√	√√	√	√
	Agenda setting and time allocation	√												√	√			√	√					
	Roles in decision-making are defined						√√	√							√		√	√	√				√	
	Independence in decision-making													√										
	Involvement since first stage of decision process									√			√	√								√		
	Involvement throughout types of decision activities	√		√	√		√			√√√	√	√√	√	√	√√		√	√	√			√		√
	Involvement throughout stages of decision process			√					√					√								√		
	Perceived influence on decision-making process		√√			√	√		√√						√			√		√	√√			
	Involvement in finalizing decisions													√										
	Control over the meeting minutes														√			√						
	Assurance of follow-up commitment/translation into action															√						√√		
	Evaluation of the decision-making process			√		√		√	√√	√			√	√		√				√	√			
	Revision process (for changing decisions or handling complaints)			√																				
**Surrogate process metrics**	^**^ **Formal power** ^a^			√	√					√	√	√		√	√	√	√	√	√					
	^**^ **Organizational commitment to engagement** ^a^		√√		√	√	√√	√	√	√√	√		√√				√√		√			√√		
	^**^ **Participation:**		√√			√	√√	√		√	√			√	√	√		√		√				√
	Activeness of participation													√										√
	Equality of participation (among P2C2 participants)		√			√	√							√						√	√			
	Attendance of engagement participants														√									
	Regularity of meetings									√					√	√		√		√				
	P2C2 participants’ readiness and attitudes towards engagement		√			√	√	√																
	^**^ **Tailoring the engagement to P2C2 participants**																√							
	Cultural beliefs and practices																√							
**Preconditions for engagement metrics**	**P2C2-initiated engagement**		√																					
	^**^ **Recruitment and membership:**			√										√	√√	√	√	√	√			√	√	
	Method of recruitment			√										√	√		√	√	√			√	√	
	Number of P2C2 members and P2C2 versus non-P2C2 participant ratio														√	√		√	√					
	Time or terms mandate for membership														√									
	^**^ **Representativeness and accountability:**		√	√		√					√			√	√		√	√	√		√	√		
	Constituent representativeness and accountability		√	√										√			√					√		
	Democratic representativeness		√											√										
	Diversity representativeness			√		√					√						√				√	√		
	^**^ **Resources provided to P2C2 participants, among them** ^a^ **:**	√√		√		√	√√		√√	√	√		√	√	√√		√	√√	√	√√	√√	√√	√	
	Support for disseminating results of the engagement					√					√		√		√			√	√			√√	√	
	Use of a broader P2C2 needs and strengths assessment to support P2C2 representatives in their decision-making			√																				
	Unbiased, jargon-free information on which to make decisions	√												√	√			√	√		√			
	^**^ **Training (for P2C2 participants)** ^a^								√√					√	√	√	√	√	√	√				
	^**^ ** Training (for staff)** ^a^									√	√		√				√					√		
**Aggregate process metrics**	**Respect**						√										√				√			
	**Transparency of the decision-making process**																				√	√	√	
	**Trust**							√													√			
	**Level of participation**													√										√
	Filled out by: External Evaluator	E		E										E		E			E				E	
	Leader^***^		L		L	L	L	L	L	L	L	L	L		L	L	L	L		L		L		L
	P2C2 representative^***^		R			R	R	R	R	R	R							R		R	R			

Abbreviation: P2C2; patient, public, consumer and community. KEY: √ 1-3 instances, √√ 4-14 instances, √√√ 15 or more instances of coding. ^*^ Row includes instances of items coded only into the outcome metric category when further specification was not possible. ^**^ Row include instances of items coded only into the subdomain when further specification was not possible. ^***^ Individual coded items could be asked of only leaders, only representatives or both; data shown are for tool as whole. ^a^ For brevity not all coded metrics are shown. For the full list of metrics, see the table in online Supplementary file 5. Some metrics were absent from all evaluation tools. These are available in Supplementary file 5.

Individual tools varied in their coverage of process metrics, ranging from one tool that included three instances in three metric subdomains to another tool that included 41 instances within six subdomains of process metrics. Seven tools included 15 or more instances of coded process metrics. Five of them were designed to be filled out by both leaders and P2C2 representatives and the remaining two by the leaders only. For those seven tools, the most frequently coded subdomains followed the same pattern identified for all the tools.

Thirteen tools included both process and outcomes metrics; three of these tools contained 4-14 instances of outcome metrics and 15 or more instances of coded process metrics. All of these three tools were designed to be filled out by both leaders and P2C2 representatives.

## Discussion

This systematic review of metrics for evaluating P2C2 engagement in healthcare organization-, community-, and system-level decision-making produced the following principal findings. First, from our qualitative analysis, we developed a comprehensive taxonomy of 116 possible engagement metrics grouped into distinct domains and subdomains. Second, we identified 23 tools that could be used to evaluate P2C2 engagement. There was no perfect tool: they varied in their coverage of the taxonomy, in the method used (ie, qualitative versus quantitative) and intended evaluators (leaders, P2C2 representatives, or both, or, alternatively, external experts). Third, parts of the metric taxonomy were absent from all tools.

### Taxonomy of Metrics

The developed taxonomy illustrates that the literature on P2C2 engagement describes a large variety of possible process and outcome metrics. Perhaps reflecting an emphasis on engagement as itself a process, nearly twice as many process metrics (72) were identified compared to outcome metrics (44). Among outcome metrics, more addressed internal outcomes (ie, the effect of P2C2 engagement on the organization itself, its services, or engagement participants) than external outcomes (ie, the effect of engagement beyond the organization where it occurs).

Among process metrics, we identified four domains of surrogate process metrics. However, analogous to health measures – where surrogate metrics may or may not evaluate the clinically significant endpoint – surrogate engagement process metrics, such as attendance or formal organizational commitment to engagement, may or may not evaluate the engagement process meaningfully.

Finally, among process metrics inquiring into preconditions necessary for engagement process, representativeness and accountability constitute a unique measurement challenge. Deciding on theoretical constructs of representativeness and representatives’ accountability and then translating those constructs into a feasible evaluation involving, if needed, the constituents themselves, will require complex multidisciplinary solutions.

For both process and outcomes metrics, aggregate metrics, such as whether P2C2 participants feel generally respected in the engagement process, may be useful as starting points but provide little insight into why this is the case or how to improve the P2C2 engagement. Similarly, evaluations employing a general ladder of participation approach^56^ (which ranges from “manipulation” of P2C2 participants by the organization to “partnership” to complete P2C2 “control” over the process) may guide an overall engagement approach but fail to provide specific actionable feedback on engagement.

Our taxonomy was developed inductively and has similarities and differences with prior taxonomies or frameworks.^57^ It similarly includes subdomains of transparency, representativeness, and resource support, and attention to P2C2 participants’ control over the decision process (including their early involvement and independence in decision-making). However, consistent with prior reviews,^27,28^ our taxonomy includes attention to evaluating outcomes of engagement and further differentiates and structures engagement process metrics. As a result, the identified metrics provide finer-grained details about both process and outcome evaluation aspects pertaining to P2C2 engagement.

### Existing Tools Measuring P2C2 Engagement

The identified tools varied methodologically and reflected greater emphasis on processes as compared to outcomes. Measuring outcomes of engagement is difficult in part because there may or may not be a proven causal connection between engagement and outcomes of interest. Consequently, many tools rely on measuring perceived benefits of engagement. However, meaningful engagement is more than mere perception; real outcomes (ie, documented changes in policies, procedures, or programs) should be preferred over perceived ones (ie, whether engagement participants or leaders believe they are making a difference). At the same time, the absence of real outcomes in tools may not be surprising; evaluating certain real outcomes (eg, cost) may not require a tool per se when this information is available by other means. Finally, several tools highlight how qualitative methods can complement the assessment of engagement process.

It is important to note metrics which are underemphasized or absent from all tools. Regarding outcomes, improved trust in the organization may be widely perceived as a potential benefit of P2C2 engagement,^58^ but it was only measured by two tools. Neither did any tool measure sustainability of engagement (understood as the ability to maintain engagement with specific P2C2 participants over time) or the capacity to increase or scale engagement in other parts of the healthcare organization or system. Regarding processes, agenda-setting and time allocation decisions, which are considered key aspects of P2C2 engagement^59^ that help mitigate power imbalances, were evaluated in only five tools, and P2C2 participants’ involvement in finalizing decisions was evaluated in only one. Debate intensity, which was proposed in the 1970s as a measure of consumer input,^60^ was completely absent.

Our findings add to prior reviews^23,27-29,61^ in important ways. Expanding the date range of our search identified additional evaluation tools. Four of the 23 tools were published in 2015, suggesting that this is an area of active research and development, and three tools were available before 1990. A notable example is Steckler and Dawson,^34^ who in 1978 published an evaluation of consumer participation in US Health Systems Agencies (HSAs). HSAs were geographically defined agencies that made decisions about healthcare planning and resource use and were legislatively required to have consumer participation.^62^ Steckler and Dawson’s evaluation relied on a structured questionnaire and interview data from both consumer participants and staff leaders.

### Practice, Policy and Research Implications

Our systematic review has implications for the implementation of P2C2 engagement and for future research. For those seeking to evaluate P2C2 engagement in real-world settings, several tools exist to get started. They vary methodologically, with some methods (eg, qualitative interviews and analysis) requiring more time, resources and expertise than others. Although, in our view, no evaluation tool is perfect and a comprehensive mapping of tools to all possible engagement contexts would be beyond the scope of this review, we were able to make three observations and practical suggestions to help potential evaluators in choosing among tools using Tables 2 and 3.

First, in certain healthcare contexts, significant emphasis may be placed on whether P2C2 engagement demonstrates positive impact via quantitatively-measured outcomes (such as improving individuals’ knowledge or improving healthcare service quality, among others). In those contexts, two tools stand out in Table 2 as being most comprehensive in the area of outcomes.^38,39^ Second, while most included tools paid significant attention to process measures, again two stood out in Table 3. One was a quantitative assessment tool^46^ used for healthcare programs and based on the decades of work from Rifkin and colleagues,^35^ and another was a more qualitative assessment designed for individual hospitals in the United States.^47^ Third, it is important to note that some tools should be used together. For instance, the Participant questionnaire and Organization questionnaire of the Public and Patient Engagement Evaluation tool together provide a mixed method evaluation that is capable of assessing the perspectives of both P2C2 representatives and organizational leaders in different contexts.^51^

**Figure 3:**
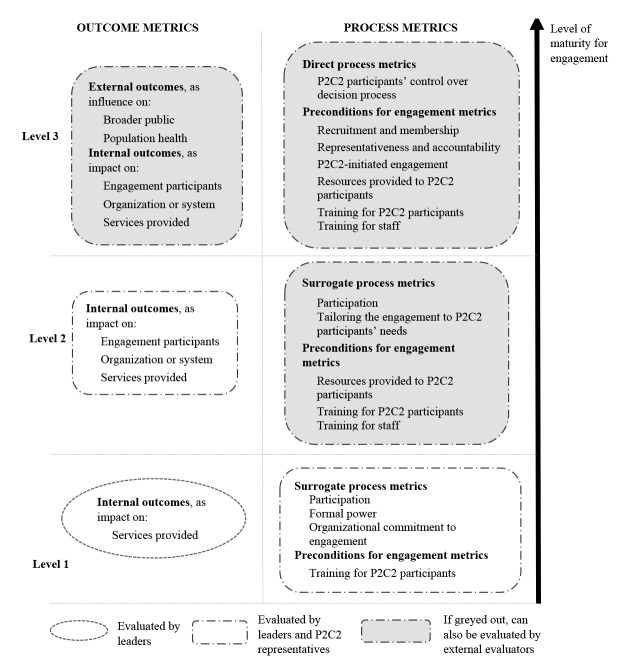
Proposed Metric Subdomains for Evaluating Patient, Consumer, Public and Community (P2C2) Engagement According to System- or Organizational Maturity for Engagement.

We can also interpret our taxonomy of metrics, which was developed inductively from the literature through the lens of Arnstein’s “ladder of participation.”^56^ This theoretical approach characterizes levels of engagement (ranging from, eg, manipulation to information to partnership to citizen control) and suggests that the goal of engagement is to increase P2C2 control over the decision process. Through Arnstein’s lens, we can suggest what may be the essential components of evaluation and how to advance evaluation over time (Figure 3).

These suggestions are tailored to a healthcare system or organization’s level of maturity for P2C2 engagement. First, although completely defining the discrete levels of maturity is beyond the scope of this paper, implicit in this figure (Figure 3) is the idea that systems or organizations should endeavor to progress from Level 1 (basic maturity, ie, ready to involve P2C2 participants) to Level 2 (intermediate maturity, ie, some experience with P2C2 engagement) to Level 3 (fully mature, ie, significant experience). Second, even though some tools identified in our review included only the system or organization leaders’ perspectives, in our view, any evaluation must solicit the P2C2 perspective on engagement, and when systems or organizations evaluate cost-effectiveness, they should include the time and resource costs accruing to P2C2 participants. Third, as organizations become more mature, relative emphasis on different process and outcome metrics changes. For instance, mature systems and organizations should shift away from surrogate measures of P2C2 participants control over decision-making toward direct control measures; likewise, they should be more attentive to external outcomes. Finally, as systems and organizations mature, they should consider using external evaluators, who could yield additional insights into the P2C2 engagement process.

From our review we suggest three priority areas of future research. First, additional research is needed to better understand the validity and reliability of tools and metrics in different healthcare contexts. The lack of evidence of rigorous testing of the identified tools for methodological quality and psychometric properties was noticeable; it suggests a need to increase testing and reporting of these factors. Second, our taxonomy and the diversity of metrics employed by the tools argue for a consensus-building process to identify and disseminate core metrics. Our taxonomy provides a starting point for that process, which itself must include P2C2 participants. As research into valid and reliable engagement metrics proceeds, attention will need to turn to comparative evaluation of the metrics (eg, to find the best way to measure representativeness) and the use of those metrics for comparative evaluation of different methods of engagement (eg, single representatives versus patient councils). Finally, new tools may be needed in order to capture parts of the taxonomy not currently represented in existing tools.

## Strengths and Limitations

This review has several strengths. The search strategy was designed to be as broad and inclusive as possible in both terminology and date range. The resulting taxonomy was constructed not from any single theoretical approach to P2C2 engagement but instead from an inductive data-driven analysis and comprehensive literature review. Moreover, the findings from this review may inform other fields of P2C2 engagement; for example, the identified process metrics might be of interest to those evaluating P2C2 engagement in research, environmental or other social policy settings.

Like all studies, ours has limitations. First, we focused on organization-, community-, and system-level decision-making in healthcare administration and organization. We excluded engagement, for instance, in other health-related fields, such as public health, health promotion or health education, and in other non-health fields. These were excluded to focus our review, and because existing engagement frameworks consider engagement in organization-, community-, and system-level decisions as a conceptually distinct activity from engagement in more societal-level decisions.^2^ While the translation of evaluation concepts and tools from these other fields could be possible and useful, that task is beyond the scope of this systematic review. Second, although our search strategy was designed to be comprehensive, certain terms (such as “health system”) may have different meanings in different countries and contexts. This could have affected our results. Moreover, terminology surrounding P2C2 engagement is imprecise (eg, patients versus consumers versus community members), and at present there appears to be no consensus regarding the aims and objectives of this type of engagement. This presents challenges to a literature search and systematic review. For instance, “community” engagement may be distinct from “patient” engagement in some circumstances (eg, when a healthcare organization wants to engage directly with a community that has historical distrust of that organization). Finally, because our review employed qualitative analysis, it may have introduced subjectivity in our categorization of metrics.

## Conclusions

Significant progress is being made in the evaluation of P2C2 engagement in healthcare organization- and system-level decision-making. The comprehensive taxonomy developed here suggests that organizations have ample process and outcome metrics as well as evaluation tools from which to choose when evaluating engagement efforts. Future research is needed to compare existing tools in practice, to develop new tools to capture all relevant metrics, and to use these tools to compare the effectiveness of different methods of engagement. This review lays the foundation for doing so.

## Acknowledgments

The work has been funded by a grant from the U.S. Agency for Healthcare Research and Quality (1K08HS023684-01). The funder did not play any role in the study design, collection, analysis, and interpretation of data, in the writing of the report; and in the decision to submit the article for publication.

## Ethical issues

Not applicable.

## Competing interests

Authors declare that they have no competing interests.

## Authors’ contributions

MD conceived of and designed the study. VD conducted the literature search, all authors performed study selection and data extraction, VD and MD synthesized the data. All authors contributed to the interpretation of the results. VD and MD were major contributors in writing the manuscript. All authors revised the manuscript, read and approved the final manuscript.

## Authors’ affiliations

^1^Department of Health Policy and Management, Johns Hopkins Bloomberg School of Public Health, Baltimore, MD, USA. ^2^National Journal, Washington, DC, USA. ^3^Johns Hopkins Berman Institute of Bioethics, Baltimore, MD, USA. ^4^Division of General Internal Medicine, Department of Medicine, Johns Hopkins School of Medicine, Baltimore, MD, USA.

## Supplementary files

Supplementary file 1. Databases search strategy.

Supplementary file 2. Gray literature sources.

Supplementary file 3. List of all publications included in the qualitative analysis to develop the taxonomy.

Supplementary file 4. Taxonomy of metrics of patient, public, consumer and community (P2C2) engagement in healthcare system-, community-, and organization-level decision-making.

Supplementary file 5. Full version of Table 3.
