# Supplementary material for: Metrics and Evaluation Tools for Patient Engagement in Healthcare Organization- and System-Level Decision-Making: A Systematic Review
Source: Int J Health Policy Manag. 2018 May 16;7(10):889–903. doi: 10.15171/ijhpm.2018.43 (PMC6186472; doi:10.15171/ijhpm.2018.43)
Supplement: Supplementary file 1 — Databases search strategy. [file ijhpm-7-889-s001.pdf]

**Supplementary File 1.** Databases search strategy

*PubMed*:

1. "Consumer Participation"[Mesh]
2. "Patient Participation"[Mesh]
3. "Patient Advocacy"[Mesh]
4. "public involvement"[Tiab], "public engagement"[Tiab], "public participation"[Tiab], "public perspective"[Tiab], "public perspectives"[Tiab], "public representation"[Tiab], "public collaboration"[Tiab], "social involvement"[Tiab], "social engagement"[Tiab], "social participation"[Tiab], "social perspective"[Tiab], "social perspectives"[Tiab], "social representation"[Tiab], "social collaboration"[Tiab], "community involvement"[Tiab], "community engagement"[Tiab], "community participation"[Tiab], "community perspective"[Tiab], "community perspectives"[Tiab], "community representation"[Tiab], "community collaboration"[Tiab], "citizen involvement"[Tiab], "citizen engagement"[Tiab], "citizen participation"[Tiab], "citizen perspective"[Tiab], "citizen perspectives"[Tiab], "citizen's participation"[Tiab], "citizen's perspective"[Tiab], "civic involvement"[Tiab], "civic engagement"[Tiab], "civic participation"[Tiab], "civic representation"[Tiab], "stakeholder involvement"[Tiab], "stakeholder engagement"[Tiab], "stakeholder participation"[Tiab], "stakeholder perspective"[Tiab], "stakeholder perspectives"[Tiab], "stakeholder representation"[Tiab], "stakeholder collaboration"[Tiab], "stakeholder's perspective"[Tiab], "family involvement"[Tiab], "family engagement"[Tiab], "family participation"[Tiab], "family perspective"[Tiab], "family perspectives"[Tiab], "family representation"[Tiab], "family's involvement"[Tiab], "family's participation"[Tiab], "family's perspective"[Tiab], "family's perspectives"[Tiab], "user involvement"[Tiab], "user engagement"[Tiab], "user participation"[Tiab], "user perspective"[Tiab], "user perspectives"[Tiab], "user representation"[Tiab], "user collaboration"[Tiab], "user's participation"[Tiab], "user's perspective"[Tiab], "user's perspectives"[Tiab], "client involvement"[Tiab], "client engagement"[Tiab], "client participation"[Tiab], "client perspective"[Tiab], "client perspectives"[Tiab], "client representation"[Tiab], "client collaboration"[Tiab], "client's involvement"[Tiab], "client's engagement"[Tiab], "client's participation"[Tiab], "client's perspective"[Tiab], "client's perspectives"[Tiab], "patient involvement"[Tiab], "patient engagement"[Tiab], "patient participation"[Tiab], "patient perspective"[Tiab], "patient perspectives"[Tiab], "patient representation"[Tiab], "patient collaboration"[Tiab], "patient's involvement"[Tiab], "patient's engagement"[Tiab], "patient's participation"[Tiab], "patient's perspective"[Tiab], "patient's perspectives"[Tiab], "patient's representation"[Tiab], "patients' perspective"[Tiab], "patients' perspectives"[Tiab], "consumer involvement"[Tiab], "consumer engagement"[Tiab], "consumer participation"[Tiab], "consumer perspective"[Tiab], "consumer perspectives"[Tiab], "consumer representation"[Tiab], "consumer collaboration"[Tiab], "consumer's involvement"[Tiab], "consumer's participation"[Tiab], "consumer's perspective"[Tiab], "customer involvement"[Tiab], "customer engagement"[Tiab], "customer participation"[Tiab], "customer perspective"[Tiab], "customer's perspective"[Tiab], "patient advocacy"[Tiab], "patient advocate"[Tiab], "patient advocates"[Tiab], "patient's advocate"[Tiab], "patient's advocates"[Tiab], "consumer advocacy"[Tiab], "consumer advocate"[Tiab], "consumer advocates"[Tiab], "community advocacy"[Tiab], "community advocate"[Tiab], "community advocates"[Tiab], "public advocacy"[Tiab], "public advocate"[Tiab], "public advocates"[Tiab], "social advocacy"[Tiab], "citizen advocacy"[Tiab], "citizen advocates"[Tiab], "family

advocacy"[Tiab], "family advocate"[Tiab], "family advocates"[Tiab], "client advocacy"[Tiab], "client advocate"[Tiab], "client advocates"[Tiab]

Terms and controlled vocabulary 1-4 were combined with the 'OR' operator, forming Block I.

5. "Decision Making, Organizational"[Mesh]
6. "Decision Making"[Mesh]
7. "Policy Making"[Mesh]
8. "Clinical Governance"[Mesh]
9. "Advisory Committees"[Mesh]
10. "Governing Board"[Mesh]
11. "advisory board"[Tiab], "advisory boards"[Tiab], "advisory committee"[Tiab], "advisory committees"[Tiab], "advisory subcommittee"[Tiab], "governing board"[Tiab], "governing boards"[Tiab], "governing committee"[Tiab], "governing committees"[Tiab], "hospital board"[Tiab], "hospital boards"[Tiab], "board of trustees"[Tiab], "hospital committee"[Tiab], "hospital committees"[Tiab], "advisory council"[Tiab], "advisory councils"[Tiab], "advisory panel"[Tiab], "advisory panels"[Tiab], "citizen panel"[Tiab], "citizen panels"[Tiab], "patient panel"[Tiab], "patient panels"[Tiab], "governance"[Tiab], "priority setting"[Tiab], "decision making"[Tiab], "decision maker"[Tiab], "decision makers"[Tiab], "policy maker"[Tiab], "policy makers"[Tiab], "policy making"[Tiab], "health care policy"[Tiab], "health care policies"[Tiab], "healthcare policy"[Tiab], "healthcare policies"[Tiab], "deliberative"[Tiab], "deliberation"[Tiab], "deliberations"[Tiab], "partnership"[Tiab], "partnerships"[Tiab], "partnering"[Tiab]

Terms and controlled vocabulary 5-11 were combined with the 'OR' operator, forming Block II.

12. "Health Care Rationing"[Mesh]
13. "Health Priorities"[Mesh]
14. "Health Care Sector"[Mesh]
15. "Health Planning"[Mesh]
16. "Accountable Care Organizations"[Mesh]
17. "health system"[Tiab], "health systems"[Tiab], "medical system"[Tiab], "medical systems"[Tiab], "health care system"[Tiab], "health care systems"[Tiab], "healthcare system"[Tiab], "healthcare systems"[Tiab], "health system"[Tiab], "health systems"[Tiab], "health organization"[Tiab], "health organizations"[Tiab], "healthcare organization"[Tiab], "healthcare organizations"[Tiab], "health care organization"[Tiab], "health care organizations"[Tiab], "medical organization"[Tiab], "medical organizations"[Tiab], "health sector"[Tiab], "health care sector"[Tiab], "healthcare sector"[Tiab], "medical sector"[Tiab], "accountable care organization"[Tiab], "accountable care organizations"[Tiab], "health care priority"[Tiab], "health care priorities"[Tiab], "healthcare priority"[Tiab], "healthcare priorities"[Tiab], "health priority"[Tiab], "health priorities"[Tiab], "hospital priority"[Tiab], "hospital priorities"[Tiab], "health care planning"[Tiab], "healthcare planning"[Tiab], "hospital planning"[Tiab], "health planning"[Tiab], "health facility planning"[Tiab], "health care rationing"[Tiab], "healthcare rationing"[Tiab], "health care distribution"[Tiab], "healthcare distribution"[Tiab], "health care administration"[Tiab], "healthcare administration"[Tiab], "hospital administration"[Tiab], "health facility administration"[Tiab], "medical administration"[Tiab], "health care resources"[Tiab], "healthcare resources"[Tiab], "hospital

resources”[Tiab], “health facility resources”[Tiab], “medical resources”[Tiab], “health care delivery”[Tiab], “healthcare delivery”[Tiab], “health care decision”[Tiab], “health care decisions”[Tiab], “healthcare decision”[Tiab], “healthcare decisions”[Tiab], “medical decision”[Tiab], “medical decisions”[Tiab], “hospital decision”[Tiab], “hospital decisions”[Tiab]

Terms and controlled vocabulary 12-17 were combined with the ‘OR’ operator, forming Block III.

Block I, Block II, and Block III were combined using the ‘AND’ operator.

*Embase*:

1. 'patient participation'/exp
2. 'consumer advocacy'/exp
3. 'patient advocacy'/exp
4. The same terms as #4 for PubMed with :ab,ti search attribute

Terms and controlled vocabulary 1-4 were combined with the ‘OR’ operator, forming Block I.

5. 'decision making'/exp
6. 'participatory management'/exp
7. 'board of trustees'/exp
8. 'advisory committee'/exp
9. The same terms as #11 for PubMed with :ab,ti search attribute

Terms and controlled vocabulary 5-9 were combined with the ‘OR’ operator, forming Block II.

10. 'health care planning'/exp
11. 'health care distribution'/exp
12. 'health care industry'/exp
13. 'accountable care organization'/exp
14. The same terms as #17 for PubMed with :ab,ti search attribute

Terms and controlled vocabulary 10-14 were combined with the ‘OR’ operator, forming Block III.

Block I, Block II, and Block III were combined using the ‘AND’ operator.

*PsycINFO, EconLit with FullText*:

1. DE "Client Participation"
2. DE "Community Involvement"
3. DE "Stakeholder"
4. DE "Advocacy"
5. The same terms as #4 for PubMed with TI and AB search attributes

Terms and controlled vocabulary 1-5 were combined with the ‘OR’ operator, forming Block I.

6. DE "Decision Making"
7. DE "Policy Making"
8. DE "Participative Management"
9. DE "Clinical Governance"
10. The same terms as #11 for PubMed with TI and AB search attributes

Terms and controlled vocabulary 6-10 were combined with the ‘OR’ operator, forming Block II.

11. DE "Health Care Administration"
12. DE "Health Care Delivery"
13. DE "Health Care Reform"
14. The same terms as #17 for PubMed with TI and AB search attributes

Terms and controlled vocabulary 7-11 were combined with the ‘OR’ operator, forming Block III.

Block I, Block II, and Block III were combined using the ‘AND’ operator.

*Sociological Abstracts*:

1. SU("Citizen Participation")
2. SU("Social Participation")
3. SU("Community Involvement")
4. SU("Advocacy")
5. The same terms as #4 for PubMed with ab,ti search attribute

Terms and controlled vocabulary 1-5 were combined with the ‘OR’ operator, forming Block I.

6. SU("Decision Making")
7. SU("Policy Making")
8. SU("Governance")
9. SU("Participative Decision Management")
10. SU("Advisory Committees")
11. SU("Governing Boards")
12. The same terms as #11 for PubMed with ab,ti search attribute

Terms and controlled vocabulary 6-12 were combined with the ‘OR’ operator, forming Block II.

13. SU("Health Planning")
14. SU("Delivery Systems")
15. SU("Medicine")
16. The same terms as #17 for PubMed with ab,ti search attribute

Terms and controlled vocabulary 13-16 were combined with the ‘OR’ operator, forming Block III.

Block I, Block II, and Block III were combined using the ‘AND’ operator.
