# Supplementary material for: Metrics and Evaluation Tools for Patient Engagement in Healthcare Organization- and System-Level Decision-Making: A Systematic Review
Source: Int J Health Policy Manag. 2018 May 16;7(10):889–903. doi: 10.15171/ijhpm.2018.43 (PMC6186472; doi:10.15171/ijhpm.2018.43)
Supplement: Supplementary file 2 — Gray literature sources. [file ijhpm-7-889-s002.pdf]

Supplementary file 2. Gray literature sources

Websites of the following organizations:

- AcademyHealth (USA)
- Agency for Healthcare Research and Quality (USA)
- America's Health Insurance Plans (USA)
- American Hospital Association (USA)
- American Institutes for Research (USA)
- Australian Institute of Health and Welfare (Australia)
- Canadian Policy Research Networks (Canada)
- Centers for Medicare & Medicaid Services (USA)
- Consumer Federation of America (USA)
- Dartmouth Institute (USA)
- European Patients Forum (International)
- Food and Drug Administration (USA)
- Health Canada (Canada)
- Healthcare Information and Management Systems Society (USA)
- Institute for Health Metrics and Evaluation (USA)
- Institute for Healthcare Improvement (USA)
- Institute for Patient- and Family-Centered Care (USA)
- Institute On Governance (Canada)
- Kellogg Foundation (USA)
- Ministry of Health (New Zealand)
- National Association of County & City Health Officials (USA)
- National Committee for Quality Assurance (USA)
- National Health Service (UK)
- National Institute for Health and Care Excellence (UK)
- National Partnership for Women & Families (USA)
- Organisation for Economic Co-operation and Development (International)
- Patient and Family Advisory Councils (USA)
- Patient-Centered Outcomes Research Institute (USA)
- Robert Wood Johnson Foundation (USA)
- Royal Pharmaceutical Society (UK)
- Scottish National Health Service (UK)
- World Health Organization (International)
