# Supplementary material for: Metrics and Evaluation Tools for Patient Engagement in Healthcare Organization- and System-Level Decision-Making: A Systematic Review
Source: Int J Health Policy Manag. 2018 May 16;7(10):889–903. doi: 10.15171/ijhpm.2018.43 (PMC6186472; doi:10.15171/ijhpm.2018.43)
Supplement: Supplementary file 3 — List of all publications included in the qualitative analysis to develop the taxonomy. [file ijhpm-7-889-s003.pdf]

Supplementary file 3. List of all publications included in the qualitative analysis to develop the taxonomy (excluding those containing P2C2 engagement evaluation tools, which are in Table 1).

Abelson, J., Montesanti, S., Li, K., Gauvin, F., & Martin, E. (2010). *Effective strategies for interactive public engagement in the development of healthcare policies and programs.* Canadian Health Services Research Foundation.

Abelson, J., Forest, P. G., Eyles, J., Smith, P., Martin, E., & Gauvin, F. P. (2003). Deliberations about deliberative methods: Issues in the design and evaluation of public participation processes. *Social Science & Medicine (1982), 57*(2), 239-251.

Abelson, J., Forest, P., Eyles, J., Casebeer, A., Martin, E., & Mackean, G. (2007). Examining the role of context in the implementation of a deliberative public participation experiment: Results from a Canadian comparative study. *Social Science & Medicine, 64*(10), 2115-2128.

Abelson, J., & Gauvin, F. (2008). *Assessing the impacts of public participation: Concepts, evidence and policy implications.* Unpublished manuscript.

Alberta Health Services Engagement and Patient Experience Department. (2014). *A resource toolkit for engaging patient and families at the planning table.* Alberta Health Services.

Alborz A., Wilkin D., & Smith K. (2002). Are primary care groups and trusts consulting local communities? *Health and Social Care in the Community, 10*(1), 20-28.

Allsop J., & Jones K. (2002). Patient involvement. Hearing voices. *Health Service Journal, 112*(5798), 28-29.

Allsop J., & Taket A. (2003). Evaluating user involvement in primary healthcare. *International Journal of Healthcare Technology and Management, 5*(1-2), 34-44.

Amaro, H., McGuire, J., Hardy-Fanta, C., & Weissman, G. (1999). Participation of latino community-based organizations in the RWCA process: A study of two metropolitan areas. *Free Inquiry in Creative Sociology, 27*(2), 29-44.

Anton S., McKee L., Harrison S., & Farrar S. (2007). Involving the public in NHS service planning. *Journal of Health, Organisation and Management, 21*(4-5), 470-483.

Armstrong, N., Herbert, G., Aveling, E., Dixon-Woods, M., & Martin, G. (2013). Optimizing patient involvement in quality improvement. *Health Expectations: An International Journal of Public Participation in Health Care & Health Policy, 16*(3), e36-e47.

Barker M., & Klopper H. (2007). Community participation in primary health care projects of the Muldersdrift health and development programme. *Curationis, 30*(2), 36-47.

Barnett, P., Tenbensel, T., Cumming, J., Clayden, C., Ashton, T., Pledger, M., & Burnette, M. (2009). Implementing new modes of governance in the New Zealand health system: An empirical study. *Health Policy, 93*(2-3), 118-127.

Bath J., & Wakerman J. (2015). Impact of community participation in primary health care: What is the evidence? *Australian Journal of Primary Health, 21*(1), 2-8.

Baum F., Sanderson C., & Jolley G. (1997). Community participation in action: An analysis of the South Australian health and social welfare councils. *Health Promotion International, 12*(2), 125-134.

Bellin, L. E., Kavaler, F., & Schwarz, A. (1972). Phase one of consumer participation in policies of 22 voluntary hospitals in New York City. *American Journal of Public Health, 62*(10), 1370-1378.

Bichmann, W., Rifkin, S. B., & Shrestha, M. (1989). Towards the measurement of community participation. *World Health Forum, 10*(3-4), 467-472.

Boivin, A., Lehoux, P., Lacombe, R., Burgers, J., & Grol, R. (2014). Involving patients in setting priorities for healthcare improvement: A cluster randomized trial. *Implementation Science: 9*, 24-5908-9-24.

Boulton, A., Simonsen, K., Walker, T., Cumming, J., & Cunningham, C. (2004). Indigenous participation in the 'new' New Zealand health structure. *Journal of Health Services Research & Policy, 9 Suppl 2*, 35-40.

Bowl, R. (1996). Involving service users in mental health services: Social services departments and the National Health Service and Community Care Act 1990. *Journal of Mental Health, 5*(3), 287-303.

Bradbury, R. C. (1972). A comprehensive health planning board of directors. *Health Services Reports, 87*(10), 905-908.

Briggs, H. E. (1996). Creating independent voices: The emergence of state wide family advocacy networks. *Journal of Mental Health Administration, 23*(4), 447-457.

Bryant J., Saxton M., Madden A., Bath N., & Robinson S. (2008). Consumer participation in the planning and delivery of drug treatment services: The current arrangements. *Drug and Alcohol Review, 27*(2), 130-137.

Buck, D. S., Rochon, D., Davidson, H., McCurdy, S., & Committee of Healthcare for the Homeless--Houston. (2004). Involving homeless persons in the leadership of a health care organization. *Qualitative Health Research, 14*(4), 513-525.

Burns, D., & Taylor, M. (2000). *Auditing community participation: An assessment handbook.* Great Britain: Policy Press.

Butterfoss, F. D. (2006). Process evaluation for community participation. *Annual Review of Public Health,* 27, 323–340.

Canadian Institutes of Health Research Partnerships and Citizen Engagement Branch. (2012). *CIHR's framework for citizen engagement.* Canadian Institutes of Health Research.

Cancer Australia and Cancer Voices Australia. (2011). *National framework for consumer involvement in cancer control.* Canberra, ACT: Cancer Australia.

Carman, K., Heeringa, J., Heil, S., Garfinkel, S., Windham, A., Gilmore, D., . . . Pathak-Sen, E. (2013). *Use of public deliberation in eliciting public input: Findings from a literature review.* Rockville, MD: Agency for Healthcare Research and Quality.

Carman, K., Maurer, M., Mallery, C., Wang, G., Garfinkel, S., Richmond, J., . . . Fratto, A. (2014). *Community forum deliberative methods demonstration: Evaluating effectiveness and eliciting public views on use of evidence.* Rockville, MD: Agency for Healthcare Research and Quality.

Carman, K. L., Dardess, P., Maurer, M., Sofaer, S., Adams, K., Bechtel, C., & Sweeney, J. (2013). Patient and family engagement: A framework for understanding the elements and developing interventions and policies. *Health Affairs, 32*(2), 223-231.

Cashin, C., Scheffler, R., Felton, M., Adams, N., & Miller, L. (2008). Transformation of the California mental health system: Stakeholder-driven planning as a transformational activity. *Psychiatric Services, 59*(10), 1107-1114.

Chafe, R., Neville, D., Rathwell, T., & Deber, R. (2008). A framework for involving the public in health care coverage and resource allocation decisions. *Healthcare Management Forum, 21*(4), 6-21.

Champeau D.A., & Shaw S.M. (2002). Power, empowerment, and critical consciousness in community collaboration: Lessons from an advisory panel for an HIV awareness media campaign for women. *Women Health, 36*(3), 31-50.

Charles, C., & DeMaio, S. (1993). Lay participation in health care decision making: A conceptual framework. *Journal of Health Politics, Policy and Law, 18*(4), 881-904.

Chesney, J. D. (1982). Strategies for building representative HSAs: The impact of legal structure. *Journal of Health Politics, Policy and Law, 7*(1), 96-110.

Chessie, K. (2009). Health system regionalization in Canada's provincial and territorial health systems: Do citizen governance boards represent, engage, and empower? *International Journal of Health Services: Planning, Administration, Evaluation, 39*(4), 705-724.

Chilaka, M. A. (2005). Ascribing quantitative value to community participation: A case study of the roll back malaria (RBM) initiative in five African countries. *Public Health, 119*(11), 987-994.

Chung, P., Grogan, C. M., & Mosley, J. E. (2012). Residents' perceptions of effective community representation in local health decision-making. *Social Science & Medicine, 74*(10), 1652-1659.

Coad, J., Flay, J., Aspinall, M., Bilverstone, B., Coxhead, E., & Hones, B. (2008). Evaluating the impact of involving young people in developing children's services in an acute hospital trust. *Journal of Clinical Nursing, 17*(23), 3115-3122.

Conklin, A., Morris, Z. S., & Nolte, E. (2010). *Involving the public in healthcare policy: An update of the research evidence and proposed evaluation framework.* Santa Monica, CA: RAND Corporation.

Conklin, A., Morris, Z., & Nolte, E. (2015). What is the evidence base for public involvement in health-care policy?: Results of a systematic scoping review. *Health Expectations: An International Journal of Public Participation in Health Care and Health Policy, 18*(2), 153-165.

Connor, H. (1999). Collaborative or chaos: A consumer perspective. *Australian & New Zealand Journal of Mental Health Nursing, 8*(3), 79-85.

Crawford, M., Rutter, D., & Thelwell, S. (2003). *User involvement in change management: A review of the literature.* London: NCCSDO.

Crawford, M. J., Rutter, D., Manley, C., Weaver, T., Bhui, K., Fulop, N., & Tyrer, P. (2002). Systematic review of involving patients in the planning and development of health care. *British Medical Journal, 325*(7375), 1263-1265.

Crowley, P., Green, J., Freake, D., & Drinkwater, C. (2002). Primary care trusts involving the community: Is community development the way forward? *Journal of Management in Medicine, 16*(4-5), 311-322.

Culyer, A. J. (2005). Involving stakeholders in healthcare decisions - the experience of the National Institute for Health and Clinical Excellence (NICE) in England and Wales. *Healthcare Quarterly, 8*(3), 56-60.

Davidson, J., Wiens, S., & Anderson, K. (2010). Creating a provincial family council to engage youth and families in child & youth mental health systems. *Journal of the Canadian Academy of Child and Adolescent Psychiatry, 19*(3), 169-175.

Davies, C., Wetherell, M., & Barnett, E. (2006). *Citizens at the centre: Deliberative participation in healthcare decision*. Bristol: Policy Press.

Davies, C., Wetherell, M., Barnett, E., & Seymour-Smith, S. (2005). *Opening the box: Evaluating the citizens council of NICE.* The Open University.

Davies, K., Gray, M., & Webb, S. A. (2014). Putting the parity into service-user participation: An integrated model of social justice. *International Journal of Social Welfare, 23*(2), 119-127.

Davis, T. R. V., & Specht, P. S. (1978). Citizen participation in community mental-health programs: A study in intergroup conflict and cooperation. *Group and Organization Studies, 3*(4), 456-466.

Dawson M.T. (2004). The role of consumer participation in Victorian primary care partnerships. *Australian Journal of Primary Health, 10*(2), 134-143.

Deng, C., & Wu, C. (2010). An innovative participatory method for newly democratic societies: The 'civic groups forum' on national health insurance reform in Taiwan. *Social Science & Medicine, 70*(6), 896-903.

Diamond B., Parkin G., Morris K., Bettinis J., & Bettesworth C. (2003). User involvement: Substance or spin? *Journal of Mental Health, 12*(6), 613-626.

Donchin M., Shemesh A.A., Horowitz P., & Daoud N. (2006). Implementation of the healthy cities' principles and strategies: An evaluation of the Israel healthy cities network. *Health Promotion International, 21*(4), 266-273.

El Ansari, W., & Phillips C.J. (2004). The costs and benefits to participants in community partnerships: A paradox? *Health Promotion Practice, 5*(1), 35-48.

European Patient's Forum. (2011). *Patient involvement in health technology assessment in Europe: Results of the EPF survey*. European Patient's Forum.

Eyre, R., & Gauld, R. (2003). Community participation in a rural community health trust: The case of Lawrence, New Zealand. *Health Promotion International, 18*(3), 189-197.

Ferreira, K., Hodges, S., & Slaton, E. (2013). The promise of family engagement: An action plan for system-level policy and advocacy. In A. McDonald Culp, & A., pp. 253-268. New York, NY, US: Springer Science + Business Media.

Flower J., & Wirz S. (2000). Rhetoric or reality? The participation of disabled people in NGO planning. *Health Policy and Planning, 15*(2), 177-185.

Frankish, C. J., Kwan, B., Ratner, P. A., Higgins, J. W., & Larsen, C. (2002). Challenges of citizen participation in regional health authorities. *Social Science & Medicine, 54*(10), 1471-1480.

Frumence, G., Nyamhanga, T., Mwangu, M., & Hurtig, A. K. (2014). Participation in health planning in a decentralised health system: Experiences from facility governing committees in the Kongwa district of Tanzania. *Global Public Health, 9*(10), 1125-1138.

Fudge, N., Wolfe, C. D. A., & McKevitt, C. (2008). Assessing the promise of user involvement in health service development: Ethnographic study. *British Medical Journal, 336*(7639), 313-317.

Gagliardi A.R., Lemieux-Charles L., Brown A.D., Sullivan T., & Goel V. (2008). Barriers to patient involvement in health service planning and evaluation: An exploratory study. *Patient Education and Counseling, 70*(2), 234-241.

Garney W.R., Drake K., Wendel M.L., McLeroy K., Clark H.R., & Ryder B. (2013). Increasing access to care for Brazos valley, Texas: A rural community of solution. *Journal of the American Board of Family Medicine, 26*(3), 246-253.

Gauvin, F., Abelson, J., Giacomini, M., Eyles, J., & Lavis, J. N. (2010). "It all depends": Conceptualizing public involvement in the context of health technology assessment agencies. *Social Science & Medicine, 70*(10), 1518-1526.

Gilbert D. (2003). Nothing about us without us: What patient and public involvement means to CHI. *Quality in Primary Care, 11*(1), 61-65.

Gilbert, D., Lloyd, P., Hampton, K., Rennie, R., & Havinden, J. (2001). *Signposts: A practical guide to public and patient involvement in Wales.* OPM/National Assembly for Wales.

Gold, S. K., Abelson, J., & Charles, C. A. (2005). From rhetoric to reality: Including patient voices in supportive cancer care planning. *Health Expectations, 8*(3), 195-209.

Grant, J., Sears, N. A., & Born, K. (2008). Public engagement and the changing face of health system planning. *Healthcare Management Forum, 21*(4), 22-26.

Greer, A. L. (1976). Training board members for health planning agencies. A review of the literature. *Public Health Reports, 91*(1), 56-61.

Gurung, G., & Tuladhar, S. (2013). Fostering good governance at peripheral public health facilities: An experience from Nepal. *Rural and Remote Health, 13*(2), 2042.

Harrington, A. D. (2008). Evaluating community participation in health care decision-making: The case of the Airdrie/North Rocky view health needs project. *Theses and Dissertations (Comprehensive).* Paper 866.

Hausner, T., Johnson, F. T., Jr, & Sevick, J. R. (1982). Factors affecting representation of minorities and the disadvantaged in health planning agencies. *Journal of Health and Human Resources Administration, 5*(2), 133-144.

Health Research & Educational Trust. (2015). *Partnering to improve quality and safety: A framework for working with patient and family advisors.* Chicago, IL: Health Research & Educational Trust.

Heller, K., Price, R. H., Reinharz, S., Riger, S., & Wandersman, A. (1984). *Psychology and community change: Challenges of the future* (Second Edition). Pacific Grove, CA: Brooks/Cole Publishing Company.

Hessler, R. M. (1977). Citizen participation, social organization, and culture: A neighborhood health center for chicanos. *Human Organization, 36*(2), 124-134.

Howard-Grabman, L. (2000). Bridging the gap between communities and service providers: Developing accountability through community mobilisation approaches. *IDS Bulletin, 31*(1), 88-96.

Howe, D., Batchelor, S., & Bochynska, K. (2011). Finding our way: Youth participation in the development and promotion of youth mental health services on the NSW Central Coast. *Advances in Mental Health, 10*(1), 20-28.

Institute for Patient- and Family-Centered Care. (2016). *Advancing the practice of patient- and family-centered care in primary care and other ambulatory settings: How to get started….* Institute for Patient- and Family-Centered Care.

Jagananen, P., & Kortenbout, E. (1999). Stimulating community participation in a group of farm workers using action research. *Curationis, 22*(1), 36-41.

Jain A. (2015). Patient communities reform healthcare in India. *British Medical Journal,* 350:h225.

Johns, S., Kilpatrick, S., & Whelan, J. (2007). Our health in our hands: Building effective community partnerships for rural health service provision. *Rural Society, 17*(1), 50-65.

Johnson A., & Silburn K. (2000). Community and consumer participation in Australian health services - an overview of organisational commitment and participation processes. *Australian Health Review, 23*(3), 113-121.

Johnson A.E., Beacham B., Moretti C., & Wishart J. (2006). Concerns about being a health consumer representative: Results of a South Australian study on consumer perspectives. *Australian Journal of Primary Health, 12*(3), 94-103.

Jolley G., Lawless A., & Hurley C. (2008). Framework and tools for planning and evaluating community participation, collaborative partnerships and equity in health promotion. *Health Promotion Journal of Australia, 19*(2), 152-157.

Jonas S. (1978). Limitations of community control of health facilities and services. *American Journal of Public Health, 68*(6), 541-543.

Kamuzora, P., Maluka, S., Ndawi, B., Byskov, J., & Hurtig, A. K. (2013). Promoting community participation in priority setting in district health systems: Experiences from Mbarali district, Tanzania. *Global Health Action, 6*, 22669.

Kapiriri, L., & Martin, D. K. (2010). Successful priority setting in low and middle income countries: A framework for evaluation. *Health Care Analysis, 18*(2), 129-147.

Kegler, M. C., Norton, B. L., & Aronson, R. E. (2008). Strengthening community leadership: Evaluation findings from the California healthy cities and communities program. *Health Promotion Practice, 9*(2), 170-179.

Kemper, C., Blackburn, C., Doyle, J. A., & Hyman, D. (2013). Engaging patients and families in system-level improvement: A safety imperative. *Nursing Administration Quarterly, 37*(3), 203-215.

Kenny, A., Farmer, J., Dickson-Swift, V., & Hyett, N. (2014). Community participation for rural health: A review of challenges. *Health Expectations.*

Kenny, A., Hyett, N., Sawtell, J., Dickson-Swift, V., Farmer, J., & O'Meara, P. (2013). Community participation in rural health: A scoping review. *BMC Health Services Research, 13*, 64-6963-13-64.

Kohler, J. C., & Martinez, M. G. (2015). Participatory health councils and good governance: Healthy democracy in Brazil? *International Journal for Equity in Health, 14*(1), 21.

Koseki, L. K. (1977). Consumer participation in health maintenance organizations. *Health & Social Work, 2*(4), 50-69.

Kovacs Burns, K., Bellows, M., Eigenseher, C., & Gallivan, J. (2014). 'Practical' resources to support patient and family engagement in healthcare decisions: A scoping review. *BMC Health Services Research, 14,* 175-6963-14-175.

Kraus F., Levy J., & Oliviere D. (2003). Brief report on user involvement at St Christopher's hospice. *Palliative Medicine, 17*(4), 375-377.

Lasker R.D., Weiss E.S., Baker Q.E., Collier A.K., Israel B.A., Plough A., & Bruner C. (2003). Broadening participation in community problem solving: A multidisciplinary model to support collaborative practice and research. *Urban Health, 80*(1), 14-60.

Leviten-Reid, C., & Hoyt, A. (2009). Community-based home support agencies: Comparing the quality of care of cooperative and non-profit organizations. *Canadian Journal on Aging, 28*(2), 107-120.

Linhorst, D. M., Eckert, A., & Hamilton, G. (2005). Promoting participation in organizational decision making by clients with severe mental illness. *Social Work, 50*(1), 21-30.

Litva, A., Canvin, K., Shepherd, M., Jacoby, A., & Gabbay, M. (2009). Lay perceptions of the desired role and type of user involvement in clinical governance. *Health Expectations, 12*(1), 81-91.

Llewellyn-Jones, L., & Harvey, D. (2005). The development of a health promotion community participation framework. *Australian Journal of Primary Health, 11*(2), 136-146.

Loewenson, R. (2000). Public participation in health systems in Zimbabwe. *IDS Bulletin, 31*(1), 14-20.

Mallery, C., Ganachari, D., Fernandez, J., Smeeding, L., Robinson, S., Moon, M., . . . Siegel, J. (2012). *Innovative methods in stakeholder engagement: An environmental scan.* Rockville, MD: Agency for Healthcare Research and Quality.

Maloff, B., Bilan, D., & Thurston, W. (2000). Enhancing public input into decision making: Development of the Calgary regional health authority public participation framework. *Family & Community Health: The Journal of Health Promotion & Maintenance, 23*(1), 66-78.

Maluka, S., Kamuzora, P., Sansebastian, M., Byskov, J., Ndawi, B., Olsen, O. E., & Hurtig, A. K. (2011). Implementing accountability for reasonableness framework at district level in Tanzania: A realist evaluation. *Implementation Science,* 6, 11-5908-6-11.

Martin, D. K., Abelson, J., & Singer, P. A. (2002). Participation in health care priority-setting through the eyes of the participants. *Journal of Health Services Research & Policy, 7*(4), 222-229.

Martin, G. P. (2008). Representativeness, legitimacy and power in public involvement in health-service management. *Social Science & Medicine, 67*(11), 1757-1765.

Maxwell C., Aggleton P., & Warwick I. (2008). Involving HIV-positive people in policy and service development: Recent experiences in England. *AIDS Care - Psychological and Socio-Medical Aspects of AIDS/HIV, 20*(1), 72-79.

McCabe, L., & Bradley, B. E. (2012). Supporting user participation in local policy development: The Fife dementia strategy. *Social Policy and Society, 11*(2), 157-169.

McDaid, S. (2009). An equality of condition framework for user involvement in mental health policy and planning: Evidence from participatory action research. *Disability & Society*, *24*(4), 461-474.

McGrow, G., & the Scottish Health Council. (2013). *Evaluating participation: A guide and toolkit for health and social care practitioners.* Healthcare Improvement Scotland.

Mclver, S. (1998). *Healthy debate? : An independent evaluation of citizens' juries in health settings*. London: King's Fund.

Meier B.M., Pardue C., & London L. (2012). Implementing community participation through legislative reform: A study of the policy framework for community participation in the Western Cape province of South Africa. *BMC International Health and Human Rights, 12*(1)

Mende S., & Roseman D. (2013). The aligning forces for quality experience: Lessons on getting consumers involved in health care improvements. *Health Affairs, 32*(6), 1092-1100.

Menon, D., & Stafinski, T. (2008). Engaging the public in priority-setting for health technology assessment: Findings from a citizens' jury. *Health Expectations, 11*(3), 282-293.

Metsch J.M., & Veney J.E. (1976). Consumer participation and social accountability. *Medical Care, 14*(4), 283-293.

Milewa T., Harrison S., Ahmad W., & Tovey P. (2002). Citizens' participation in primary healthcare planning: Innovative citizenship practice in empirical perspective. *Critical Public Health, 12*(1), 39-53.

Ministry of Health. (2003). *Toward clinical excellence: A toolkit to develop consumer participation in credentialling.* Wellington: Ministry of Health.

Mitton, C., Smith, N., Peacock, S., Evoy, B., & Abelson, J. (2009). Public participation in health care priority setting: A scoping review. *Health Policy, 91*(3), 219-228.

Molnar C. (2001). Addressing challenges, creating opportunities: Fostering consumer participation in Medicaid and children's health insurance managed care programs. *Journal of Ambulatory Care Management, 24*(3), 61-67.

Mosquera, M., Zapata, Y., Lee, K., Arango, C., & Varela, A. (2001). Strengthening user participation through health sector reform in Colombia: A study of institutional change and social representation. *Health Policy and Planning, 16 Suppl 2,* 52-60.

Murray, Z. (2015). Community representation in hospital decision making: A literature review. *Australian Health Review, 39,* 323-328.

Nagel, J. H. (1992). Combining deliberation and fair representation in community health decisions. *University of Pennsylvania Law Review, 140*(5), 1965-1985.

Nathan S., Harris E., Kemp L., & Harris-Roxas B. (2006). Health service staff attitudes to community representatives on committees. *Journal of Health, Organisation and Management, 20*(6), 551-559.

North, N., & Werko, S. (2002). Widening the debate? Consultation and participation in local health care planning in the English and Swedish health services. *International Journal of Health Services, 32*(4), 781-798.

Oakley, P. (1991). *Projects with people: The practice of participation in rural development*. Geneva: International Labour Office.

O'Keefe E., & Hogg C. (1999). Public participation and marginalized groups: The community development model. *Health Expectations, 2*(4), 245-254.

O'Mara-Eves, A., Brunton, G., McDaid, D., Oliver, S., Kavanagh, J., Jamal, F., . . . Thomas, J. (2013). Community engagement to reduce inequalities in health: A systematic review, meta-analysis and economic analysis. *Public Health Research, 1*(4)

O'Meara, W. P., Tsofa, B., Molyneux, S., Goodman, C., & McKenzie, F. E. (2011). Community and facility-level engagement in planning and budgeting for the government health sector--a district perspective from Kenya. *Health Policy, 99*(3), 234-243.

O'Neill, M. (1992). Community participation in Quebec's health system: A strategy to curtail community empowerment? *International Journal of Health Services, 22*(2), 287-301.

Organisation for Economic Co-operation and Development. (2005). *Evaluating public participation in policy making.* OECD.

Osher, T. W., Penn, M., & Spencer, S. A. (2008). Partnerships with families for family-driven systems of care. In B. A. Stroul, G. M. Blau, B. A., pp. 249-273. Baltimore, MD, US: Paul H Brookes Publishing.

Oxman, A. D., Lewin, S., Lavis, J. N., & Fretheim, A. (2009). SUPPORT tools for evidence-informed health policymaking (STP) 15: Engaging the public in evidence-informed policymaking. *Health Research Policy and Systems, 7 Suppl 1*, S15-4505-7-S1-S15.

Patient-Centered Outcomes Research Institute. (2014). *Ways of engaging- ENgagement ACtivity tool (WE-ENACT) – researcher 2.0 item pool.* PCORI.

Patient-Centered Outcomes Research Institute. (2015). *PCORI evaluation framework 2.0.* PCORI.

Perlstadt, H., Jackson-Elmoore, C., Freddolino, P. P., & Sturdevant Reed, C. (1999). Citizen participation in health planning: A case study of changing delivery systems. *Research in the Sociology of Health Care, 16*, 75-98.

Pickin, C., Popay, J., Staley, K., Bruce, N., Jones, C., & Gowman, N. (2002). Developing a model to enhance the capacity of statutory organisations to engage with lay communities. *Journal of Health Services Research and Policy, 7*(1), 34-42.

Ponte, P. R., Conlin, G., Conway, J. B., Grant, S., Medeiros, C., Nies, J., . . . Conley, K. (2003). Making patient-centered care come alive: Achieving full integration of the patient's perspective. *The Journal of Nursing Administration, 33*(2), 82-90.

Popay, J., Whitehead, M., Carr-Hill, R., Dibben, C., Dixon, P., Halliday, E., . . . Walthery, P. (2015). The impact on health inequalities of approaches to community engagement in the new deal for communities regeneration initiative: A mixed-methods evaluation. *Public Health Research, 3*(12)

Preston, R., Waugh, H., Larkins, S., & Taylor, J. (2010). Community participation in rural primary health care: Intervention or approach? *Australian Journal of Primary Health, 16*(1), 4-16.

Principles of community engagement (2011). (Second edition). Bethesda, MD: National Institutes of Health.

Quantz, D., & Thurston, W. E. (2006). Representation strategies in public participation in health policy: The aboriginal community health council. *Health Policy, 75*(3), 243-250.

Ramiro L.S., Castillo F.A., Tan-Torres T., Torres C.E., Tayag J.G., Talampas R.G., & Hawken L. (2001). Community participation in local health boards in a decentralized setting: Cases from the Philippines. *Health Policy and Planning, 16*, 61-69.

Rasanathan, K., Posayanonda, T., Birmingham, M., & Tangcharoensathien, V. (2012). Innovation and participation for healthy public policy: The first national health assembly in Thailand. *Health Expectations, 15*(1), 87-96.

Rawlins, M. D. (2005). Pharmacopolitics and deliberative democracy. *Clinical Medicine, 5*(5), 471-475.

Riddick, C. C., Eisele, T., & Montgomery, A. (1984). The health planning process: Are consumers really in control? *Health Policy, 4*(2), 117-127.

Rodwin, M. (2000). *Promoting accountable managed health care: The potential role for consumer voice.* Center for Law and Health, Indiana University Law School-Indianapolis.

Ruano, A. L., Sebastián, M. S., & Hurtig, A. (2014). The process of social participation in primary health care: The case of Palencia, Guatemala. *Health Expectations, 17*(1), 93-103.

Rummery, K. (2009). Healthy partnerships, healthy citizens? An international review of partnerships in health and social care and patient/user outcomes. *Social Science & Medicine, 69*(12), 1797-1804.

Rutter D., Manley C., Weaver T., Crawford M.J., & Fulop N. (2004). Patients or partners? Case studies of user involvement in the planning and delivery of adult mental health services in London. *Social Science and Medicine, 58*(10), 1973-1984.

Sabin, J. A., & Daniels, N. (2002). Managed care: Strengthening the consumer voice in managed care: V. helping professionals listen. *Psychiatric Services, 53*(7), 805-811.

Schwartz, J. L. (1964). Participation of consumers in prepaid health plans. *Journal of Health and Human Behavior, 5*, 74-84.

Serapioni, M., & Duxbury, N. (2014). Citizens' participation in the Italian health-care system: The experience of the mixed advisory committees. *Health Expectations, 17*(4), 488-499.

Shepperd, J. D. (1977). Consumer participation in community health programs: A comparative analysis of two programs. *Journal of the National Medical Association*, *69*(2), 115-119.

Shinn, C. *Meaningful community voice: Advocacy, accountability and autonomy in community health partnerships*. Ph.D. Dissertation, Heller School for Social Policy and Management, Brandeis Univ.

Sibbald, S. L., Singer, P. A., Upshur, R., & Martin, D. K. (2009). Priority setting: What constitutes success? A conceptual framework for successful priority setting. *BMC Health Services Research, 9*, 43-6963-9-43.

Sibley Memorial Hospital. (2013). *Patient and family advisory council (PFAC) annual report 2013.* Sibley Memorial Hospital.

Silvestre, A. J., Faber, J. F., Shankle, M. D., & Kopelman, J. P. (2002). A model for involving youth in health planning: HIV prevention in Pennsylvania. *Perspectives on Sexual and Reproductive Health, 34*(2), 91-97.

Sitzia J., Cotterell P., & Richardson A. (2006). Interprofessional collaboration with service users in the development of cancer services: The cancer partnership project. *Journal of Interprofessional Care, 20*(1), 60-74.

South Australian Community Health Research Unit. (2015). *Community participation evaluation tool.* SACHRU.

Steckler, A., Dawson, L., Dellinger, N., & Williams, A. (1981). Consumer participation and influence in a health systems agency. *Journal of Community Health, 6*(3), 181-193.

Sullivan M.J.L., & Scattolon Y. (1995). Health policy planning: A look at consumer involvement in Nova Scotia. *Canadian Journal of Public Health, 86*(5), 317-320.

Tataw, D. B. (2012). Toward a horizontal participatory implementation approach for community health programs serving vulnerable populations. *Journal of Human Behavior in the Social Environment, 22*(4), 421-435.

Tenbensel, T. (2002). Interpreting public input into priority-setting: The role of mediating institutions. *Health Policy, 62*(2), 173-194.

Thurston, W. E., MacKean, G., Vollman, A., Casebeer, A., Weber, M., Maloff, B., & Bader, J. (2005). Public participation in regional health policy: A theoretical framework. *Health Policy, 73*(3), 237-252.

Turan, J. M., Say, L., Gungor, A. K., Demarco, R., & Yazgan, S. (2003). Community participation for perinatal health in Istanbul. *Health Promotion International, 18*(1), 25-32.

Vincent-Jones P. (2011). Embedding economic relationships through social learning? The limits of patient and public involvement in healthcare governance in England. *Journal of Law and Society, 38*(2), 215-244.

Warburton, D., Wilson, R., & Rainbow, E. (2007). *Making a difference: A guide to evaluating public participation in central government.* The Involve Foundation.

Wasylenko E. (2013). Jugglers, tightrope walkers, and ringmasters: Priority setting, allocation, and reducing moral burden. *Healthcare Management Forum, 26*(2), 77-81.

Wilhelm, A., & Jackson, K. (2012). *All aboard for patient engagement: A resource kit for patients, providers and leaders.* Alberta Health Services.

Willcox J., & Gill M. (2007). Integrated disease management programs: Reflections and learnings from implementation. *Australian Journal of Primary Health, 13*(2), 113-120.

Winder, E., the Value+ Steering Group, Bedlington, N., & Megas, E. (2013). *The value+ toolkit: For patient organisations on meaningful patient involvement.* European Patients' Forum.

World Health Organization. Division of Mental Health. (1989). *Consumer involvement in mental health and rehabilitation services.* Geneva: World Health Organization.

Worley, C. G., Mohrman, S. A., & Nevitt, J. A. (2011). Large group interventions: An empirical field study of their composition, process, and outcomes. *Journal of Applied Behavioral Science, 47*(4), 404-431.

Young, R. (2006). Introducing role and service changes in health and social care: The impact and influence of user involvement in England and Wales. *Social Policy and Society, 5*(2), 249-268.

Zuidgeest M., Luijkx K.G., Westert G.P., & Delnoij D.M. (2011). Legal rights of client councils and their role in policy of long-term care organisations in the Netherlands. *BMC Health Services Research, 11*, 215.
