# Supplementary material for: Metrics and Evaluation Tools for Patient Engagement in Healthcare Organization- and System-Level Decision-Making: A Systematic Review
Source: Int J Health Policy Manag. 2018 May 16;7(10):889–903. doi: 10.15171/ijhpm.2018.43 (PMC6186472; doi:10.15171/ijhpm.2018.43)
Supplement: Supplementary file 4 — Taxonomy of metrics of patient, public, consumer and community (P2C2) engagement in healthcare system-, community-, and organization-level decision-making. [file ijhpm-7-889-s004.pdf]

**Supplementary file 4.** Taxonomy of metrics of patient, public, consumer and community (P2C2) engagement in healthcare system-, community-, and organization-level decision-making

| **OUTCOME METRICS** | |
|---|---|
| **Internal Outcomes**, as measured by **impact on**:<br>Engagement participants' (patients, staff, others)<br>Knowledge<br>Skills<br>Views<br>Confidence or self-esteem<br>Empowerment*<br>Satisfaction<br>Sense of ownership<br>Trust**<br>Services provided (by the healthcare organization or system)<br>Efficiency and cost-effectiveness of services<br>Number of complaints on services<br>Service availability<br>Services quality and safety<br>Services responsiveness to needs (including needs of subpopulations)<br>Sustainability of the services<br>User experiences with services<br>Utilization of services<br>Organization or system (eg, resources, policies, procedures, staff)<br>Additional potential connections or partnerships with other groups or organizations<br>Diversity of funding sources<br>Funding and resources availability<br>Visibility of organization<br>Accountability of organization to the P2C2 served***<br>Staff views on engagement<br>Staff satisfaction<br>Informal (unwritten) organization or system procedures<br>Formal (written) organization or system policies<br>Redesign of staff roles<br>Staff recruitment<br>Staff training<br>Explicit change to organization or system process of decision-making<br>Presence of racism in system<br>Level of public reporting (eg, making annual report available to the public) | **External Outcomes**, as measured by **influence on:**<br>Broader public's (outside the organization or system)<br>Capacity for future involvement in the organization by the community<br>Level of control over decisions made by the organization or system<br>Awareness or knowledge of health issues<br>Support of the organization or system<br>Involvement as part of social change outside the organization****<br>Stigmatization of others<br>Population health<br>Population health status<br>Level of health inequalities<br><br>**Aggregate Outcome:**<br>Overall cost-effectiveness of engagement |

Number of local employment positions supported by organization
Organizational ability to adapt to operating environment
Sustainability of the engagement initiative
Scale of engagement program by organization (eg, to new locations)

**PROCESS METRICS**

**Direct Process Metrics**

P2C2 participants control over decision process
- Agenda setting and time allocation
- Roles in decision-making are defined
- Independence in decision-making (ie, no organization or system constraints on decisions)†
- Involvement since first stage of decision process
- Involvement throughout types of decision activities
- Involvement throughout the stages of decision process
- Perceived influence on decision-making process
- Involvement in finalizing decisions
- Control over the meeting minutes
- Assurance of follow-up commitment / translation into action
- Evaluation of the decision-making process
- Revision process (for changing decisions or handling complaints)

**Surrogate Process Metrics**

Formal power
- Financial independence of P2C2 participants
- P2C2 participants hold formal positions within the organizational hierarchy
- P2C2 participants are protected from organization retaliation
- Statement of formal self-governance by P2C2 participants
- P2C2 participants have veto power in decision-making

Organizational commitment to engagement
- Formal declaration of support by the organization or system
- Formal expression of commitment by organization or system leadership
- Organization has a paid position(s) dedicated to engagement
- Organization or system rewards staff participation in engagement
- Staff readiness and attitudes towards engagement
- Staff have formal job responsibilities related to engagement

Participation
- Activeness of participation††
- Debate intensity (ie, a measure of the amount or intensity of debate during meetings)†††
- Equality of participation among P2C2 participants
- Attendance of engagement participants

**Preconditions for Engagement Metrics**

P2C2-initiated engagement (in contrast with mandated)

Recruitment and Membership
- Consistency of membership (turnover)
- Method of recruitment
- Number of P2C2 members and P2C2 versus non-P2C2 participant ratio
- Participants' neutrality (ie, no conflict of interest)
- Time or terms mandate for membership
- Literacy of P2C2 participants (ie, ability to read and write)

Representativeness and accountability
- Constituent representativeness and accountability (ie, represent values, needs, etc. of the relevant constituency and are accountable to that constituency)
- Democratic representativeness (ie, elected via a democratic procedure by a broader community)
- Representative legitimacy (non-democratic)††††
- Diversity representativeness (ie, minority, vulnerable or marginalized groups are represented)

Resources provided to P2C2 participants
- Accommodations (ie, lodging)
- Childcare
- Financial support
- Meals and refreshments
- Transportation and parking
- Translation support
- Meeting facilitator
- Meeting place tailored to P2C2 participants
- Meeting time tailored to P2C2 participants
- Support from other P2C2 participants
- Support from patient advocacy groups or organizations
- Support for disseminating results of the engagement
- Staff support of P2C2 involvement in engagement
- Feedback from P2C2 complaints
- Use of a broader P2C2 needs and strengths assessment to support P2C2 representatives in their decision-making

Training for P2C2 participants
- Presence of training
- Quality of training
- Scope of training
- Training provided is independent of the organization or system (in content or financing)

Training for staff
- Presence of training
- Quality of Training

| | |
|---|---|
| Attendance of P2C2 participants who are minorities | Unbiased, jargon-free information on which to make decisions |
| Regularity of meetings | **Aggregate Process Metrics** |
| P2C2 participants' readiness and attitudes towards engagement | Respect (ie, engagement participants are treated respectfully / not intimidated)††††† |
| Substitution of P2C2 participants among attendees | Transparency of the decision-making process |
| Tailoring the engagement to participants (needs or beliefs) | Trust†††††† |
| Cultural beliefs and practices | Legitimacy of decision-making |
| Individual mental health needs | Level or ladder of participation (eg, Information, Consultation, Delegation, Partnership) |
| Individual health needs | |

Clarifying examples:

* An example of an item measuring empowerment: "I learn things from the PFAC [Patient and Family Advisory Council] meetings that help me understand how to help the hospital change and improve."[1]

** An example of an item measuring trust: "As a result of my participation in this activity, I have greater trust in [administering organization to insert relevant term, eg, providers, PPE [Public and patient engagement] staff, organization as a whole, health system, personal competency]."[2]

*** An example of metric description of accountability of organization to P2C2 served: "Public had a role in ensuring that communities were protected and concerns heard when dealing with poor performance. They felt the type of involvement needed was for someone to oversee the process and feedback to the community, thus ensuring that problems were dealt with openly and ensuring greater accountability. Decisions are scrutinized by a member of the public who ensures the concerns and values of either the wider community (acting as a citizen) or the specific community (acting as an advocate) are examined and professionals held to account."[3]

**** An example of metric description of involvement as part of social change outside the organization: "Participation was not just a tool for recognising their right to involvement. Their goal was structural change. Service-user participation, though flawed, offered opportunities to take part in society and was therefore a starting point. However, the end to which service users aspired was social justice."[4]

† An example of an item description measuring independence of decision-making: "the community defines priorities and manages the program."[5]

†† An example of metric description of activeness of participation: "Do consumer members take the lead in raising issues? Are they aggressive in seeking answers to problems? Do they follow up? Are they apathetic?"[6]

††† An example of metric description of debate intensity: the Debate Intensity variable "captured a qualitative aspect," "specifically the extent to which disagreements were passionate, deep, or intense." Researcher analyzed "the amount of words spoken, the emotional intensity implied by different words" and "the extent to which perspectives expressed by one person were agreed to or disputed by another."[7]

†††† An example of metric description of representative legitimacy (non-democratic): "professionals ascribed a certain degree of representative legitimacy to involved users, on the basis of their laity (vis-a` -vis clinical expertise) and their patienthood."[8]

††††† An example of an item measuring respect (ie, engagement participants are treated respectfully / not intimidated): "Respondents were asked how often they felt intimidated by consumer board members and provider board members. Response categories were "all of the time," "most of the time," "some of the time," and "never."[9]

†††††† An example of metric description of trust: "Trust is one party's willingness to be vulnerable to another based on the belief that the latter party is competent, open, concerned and reliable. Probably the key condition for trust in the process is public and stakeholder acceptance of the policy substance that is produced by the process."[10]

**References** (for online supplemental material 4)


1. Brigham and Women's Hospital Center for Patients and Families. *Patient and Family Advisory Council (PFAC) Report Submitted September 30, 2014*. https://www.hcfama.org/sites/default/files/brigham_and_womens_dph_report_sept_2014.pdf.
2. Abelson J, PPEET Research-Practice Collaborative. *The Public and Patient Engagement Evaluation Tool*. https://fhs.mcmaster.ca/publicandpatientengagement/ppeet.html.
3. Litva A, Canvin K, Shepherd M, Jacoby A, Gabbay M. Lay perceptions of the desired role and type of user involvement in clinical governance. *Health Expect* 2009;12(1):81-91. doi:10.1111/j.1369-7625.2008.00530.x.
4. Davies K, Gray M, Webb SA. Putting the parity into service-user participation: An integrated model of social justice. *Int J Soc Welf* 2014;23(2):119-127. doi:10.1111/ijsw.12049.
5. Draper AK, Hewitt G, Rifkin S. Chasing the dragon: Developing indicators for the assessment of community participation in health programmes. *Soc Sci Med* 2010;71(6):1102-1109. doi: 10.1016/j.socscimed.2010.05.016.
6. Bellin LE, Kavaler F, Schwarz A. Phase one of consumer participation in policies of 22 voluntary hospitals in New York City. *Am J Public Health* 1972;62(10):1370-1378.
7. Worley CG, Mohrman SA, Nevitt JA. Large group interventions: An empirical field study of their composition, process, and outcomes. *J Appl Behav Sci* 2011;47(4):404-431. doi:10.1177/0021886311410837.
8. Martin GP. Representativeness, legitimacy and power in public involvement in health-service management. *Soc Sci Med* 2008;67(11):1757-1765. doi:10.1016/j.socscimed.2008.09.024.
9. Riddick CC, Eisele T, Montgomery A. The health planning process: are consumers really in control? *Health Policy* 1984;4(2):117-127.
10. Tenbensel T. Interpreting public input into priority-setting: the role of mediating institutions. *Health Policy* 2002;62(2):173-194.
