# Supplementary material for: Metrics and Evaluation Tools for Patient Engagement in Healthcare Organization- and System-Level Decision-Making: A Systematic Review
Source: Int J Health Policy Manag. 2018 May 16;7(10):889–903. doi: 10.15171/ijhpm.2018.43 (PMC6186472; doi:10.15171/ijhpm.2018.43)
Supplement: Supplementary file 5 — Full version of Table 3. [file ijhpm-7-889-s005.pdf]

Supplementary file 5

Table 3. Coding Frequencies of Process Metrics in the Taxonomy per Evaluation Tool (Full version)

	Metric	Tool (for number correspondence, see Table 1)																						
		1	2	3	4	5	6	7	8	9	10	11	12	13	14	15	16	17	18	19	20	21	22	23
	*** Process metric**	√√	√√	√√	√√	√√	√√√	√√	√√√	√√√	√	√√	√√√	√	√√√	√√	√√	√√√	√√	√√√	√√	√√	√√	√
**Direct process metrics**	**** P2C2 participants control over decision process:**	√	√√	√	√	√	√	√√	√√√	√√√	√	√	√√	√	√	√	√	√√	√	√	√√	√√	√	√
	Agenda setting and time allocation	√												√	√			√	√					
	Roles in decision-making are defined						√√	√							√		√	√	√				√	
	Independence in decision-making													√										
	Involvement since first stage of decision process									√			√	√								√		
	Involvement throughout types of decision activities	√		√	√		√			√√√	√	√√	√	√	√√		√	√	√			√		√
	Involvement throughout stages of decision process			√					√					√								√		
	Perceived influence on decision-making process		√√			√	√		√√						√			√		√	√√			
	Involvement in finalizing decisions													√										
	Control over the meeting minutes														√			√						
	Assurance of follow-up commitment / translation into action															√						√√		
	Evaluation of the decision-making process			√		√		√	√√	√			√	√		√				√	√			
	Revision process (for changing decisions or handling complaints)			√																				
**Surrogate process metrics**	**** Formal power**			√	√					√	√	√		√	√	√	√	√	√					
	Financial independence of P2C2 participants										√			√										
	P2C2 participants hold formal positions within the organizational hierarchy			√								√		√	√	√		√	√					
	Statement of formal self-governance by P2C2 participants			√										√				√						
	**** Organizational commitment to engagement**		√√		√	√	√√	√	√	√√	√		√√				√√		√			√√		
	Formal declaration of support by the organization or system					√	√			√			√									√√		
	Formal expression of commitment by organization or system leadership		√			√	√	√		√√			√						√					
	Organization has a paid position(s) dedicated to engagement									√			√											

	Metric	Tool (for number correspondence, see Table 1)																						
		1	2	3	4	5	6	7	8	9	10	11	12	13	14	15	16	17	18	19	20	21	22	23
	Organization or system rewards staff participation in engagement									√														
	Staff readiness and attitudes towards engagement		√		√	√			√	√	√						√					√		
	Staff have formal job responsibilities related to engagement																					√		
	** **Participation:**		√√			√	√√	√		√	√			√	√	√		√		√				√
	Activeness of participation													√										√
	Equality of participation (among P2C2 participants)		√			√	√							√						√	√			
	Attendance of engagement participants														√									
	Regularity of meetings									√					√	√		√		√				
	P2C2 participants’ readiness and attitudes towards engagement		√			√	√	√																
	** **Tailoring the engagement to P2C2 participants**																√							
	Cultural beliefs and practices																√							
**Preconditions for engagement metrics**	**P2C2-initiated engagement**		√																					
	** **Recruitment and membership:**			√										√	√√	√	√	√	√			√	√	
	Method of recruitment			√										√	√		√	√	√			√	√	
	Number of P2C2 members and P2C2 versus non-P2C2 participant ratio														√	√		√	√					
	Time or terms mandate for membership														√									
	** **Representativeness and accountability:**		√	√		√					√			√	√		√	√	√		√	√		
	Constituent representativeness and accountability		√	√										√			√					√		
	Democratic representativeness		√											√										
	Diversity representativeness			√		√					√						√				√	√		

	Metric	Tool (for number correspondence, see Table 1)																						
		1	2	3	4	5	6	7	8	9	10	11	12	13	14	15	16	17	18	19	20	21	22	23
	** **Resources provided to P2C2 participants**	√√		√		√	√√		√√	√	√		√	√	√√		√	√√	√	√√	√√	√√	√	
	Childcare														√		√	√	√		√	√		
	Financial support					√	√							√	√		√	√	√			√		
	Meals and refreshments																		√			√		
	Transportation and parking						√								√		√	√	√		√	√		
	Translation support																		√			√		
	Meeting place tailored to P2C2 participants																		√			√		
	Meeting time tailored to P2C2 participants																			√		√		
	Support from other P2C2 participants								√						√		√	√				√		
	Support for disseminating results of the engagement					√					√		√		√			√	√			√√	√	
	Staff support of P2C2 involvement in engagement	√					√		√√	√				√	√		√	√		√		√		
	Use of a broader P2C2 needs and strengths assessment to support P2C2 representatives in their decision-making			√																				
	Feedback from P2C2 complaints																						√	
	Unbiased, jargon-free information on which to make decisions	√												√	√			√	√		√			
	** **Training (for P2C2 participants)**								√√					√	√	√	√	√	√	√				
	Presence of training													√		√	√							
	Quality of training								√√						√	√								
	Scope of training														√	√			√					
	** **Training (for staff)**									√	√		√				√					√		
	Quality of training																					√		
**Aggregate process**	**Respect**						√										√				√			
	**Transparency of the decision-making process**																				√	√	√	
	**Trust**							√													√			
	**Level of participation**													√										√
Filled out by:	External Evaluator	E		E										E		E			E				E	
	Leader***		L		L	L	L	L	L	L	L	L	L		L	L	L	L		L		L		L
	P2C2 representative***		R			R	R	R	R	R	R							R		R	R			

P2C2 - patient, public, consumer and community.

KEY: √ 1-3 instances, √√ 4-14 instances, √√√ 15 or more instances of coding.
***** Row includes instances of items coded only into the outcome metric category when further specification was not possible.
****** Row include instances of items coded only into the subdomain when further specification was not possible.
******* Individual coded items could be asked of only leaders, only representatives or both; data shown are for tool as whole.

Metrics absent from all tools include:

- Formal power: 1) P2C2 participants are protected from organization retaliation; 2) P2C2 participants have veto power in decision-making.
- Participation: 1) debate intensity; 2) attendance of P2C2 participants who are minorities; 3) substitution of P2C2 participants among attendees.
- Tailoring the engagement to P2C2 participants (needs or beliefs): 1) individual mental health needs; 2) individual health needs.
- Recruitment and membership: 1) consistency of membership (turnover); 2) participants' neutrality; 3) literacy of P2C2 participants.
- Representativeness and accountability: representative legitimacy (non-democratic).
- Resources provided to P2C2 participants: 1) accommodations; 2) meeting facilitator; 3) support from patient advocacy groups or organizations.
- Training for P2C2 participants: training provided is independent of the organization or system (in content or financing).
- Legitimacy of decision-making (an individual subdomain under aggregate process metrics domain.
